# Diagnostik und chirurgische Therapie der Schilddrüsenerkrankungen

**DOI:** 10.1007/s00106-025-01620-5

**Published:** 2025-04-09

**Authors:** Thomas Verse, Ansgar Michael Chromik

**Affiliations:** 1 Abteilung für HNO-Heilkunde, Kopf- und Halschirurgie, Asklepios Klinikum Harburg, Eißendorfer Pferdeweg 52, 21075 Hamburg, Deutschland; 2 Universitätsklinik für HNO-Heilkunde, Kopf- und Halschirurgie Universitätsmedizin Mannheim, Universität Heidelberg, Theodor-Kutzer-Ufer 1–3, 68167 Mannheim, Deutschland; 3 Abteilung für Allgemein- und Viszeralchirurgie, Asklepios Klinikum Harburg, Eißendorfer Pferdeweg 52, 21075 Hamburg, Deutschland

**Keywords:** Anatomie, Physiologie, Schilddrüsenerkrankungen, Allgemeinchirurgie, Komplikationen, Anatomy, Physiology, Thyroid gland disorders, General surgery, Complications

## Abstract

Das Interesse der HNO-Heilkunde an der Schilddrüse steigt. Das vorliegende Referat gibt einen Überblick als Einstiegshilfe in das Thema. Zuerst werden die chirurgische Anatomie und die Physiologie dargestellt. Im Weiteren werden die häufigsten benignen und malignen Erkrankungen der Schilddrüse skizziert. Der Fokus der Darstellung der Therapie liegt auf der operativen Therapie mit all ihren perioperativen Aspekten und ihren Komplikationen und deren Management.

**Zusatzmaterial online:**

Die Online-Version dieses Artikels (10.1007/s00106-025-01620-5) enthält weiterführende Literatur.

## Abstract (en)

There is a growing interest in diseases of the thyroid gland within the ENT community. This review provides an introduction to the topic. The first section describes the surgical anatomy and physiology of the thyroid gland. Subsequently, the most frequent benign and malignant diseases of the thyroid gland are discussed. Regarding treatment of thyroid diseases, the focus is placed on surgical therapy with all its perioperative issues as well as surgical complications and their management.

Der diesjährige Jahreskongress der Deutschen Gesellschaft für Hals‑, Nasen‑, Ohrenheilkunde, Kopf- und Halschirurgie steht unter dem Motto Individualisierung versus Standardisierung. Beides beeinflusst unser tägliches klinisches Wirken. Bei der individualisierten Medizin gehen der Behandlung diagnostische Tests voraus, deren Ergebnis eine auf den Patienten und sein Krankheitsbild passgenau abgestimmte Therapie oder auch Prävention ermöglichen soll. So wünschenswert dieser Ansatz ist, so stehen ihm gesundheitsökonomische Zwänge entgegen. Ein „alles für jeden“ ist schon lange nicht mehr finanzierbar. Standardisierung und Konzentration sind aktuell bevorzugte Mittel für die Effizienzbemühungen in der Medizin vor dem Problem der Endlichkeit der Finanzmittel. Um diesem Spannungsfeld auch künftig gerecht werden zu können, bedarf es eines guten Fachwissens. Das gilt insbesondere dann, wenn man sich an den Rand des eigenen Fachgebiets begibt. Die Schilddrüse ist für den HNO‑Arzt ein solches Organ, welches zwar im Hals liegt, aber zumindest in Deutschland auch von anderen Fachgebieten bedient wird. Was die Schilddrüsenchirurgie anbelangt, haben die Autoren ihre Bemühungen zusammengelegt, statt miteinander zu konkurrieren. Dabei waren unterschiedliche Herangehensweisen an die Schilddrüse festzustellen, sodass beide Seiten sehr – nämlich im Sinne von „das Beste aus 2 Welten“ voneinander profitieren. Versuchen Sie es auch! Wir bieten Ihnen nachstehend einen gemeinsamen Blick von chirurgischer und HNO-ärztlicher Seite auf die Schilddrüse.

## Chirurgische Anatomie

Die Schilddrüse ist schmetterlingsförmig und sitzt im unteren mittleren Halsanteil auf der Trachea, mit der sie beidseits bindegewebig („Berry’s ligament“) verbunden ist. Dadurch folgt die Schilddrüse beim Schluckakt den Bewegungen des Kehlkopfs. Die Palpation der Schilddrüse erfolgt daher auch während des Schluckakts. Die Schilddrüse wird in einen rechten und einen linken Anteil unterteilt, der prätracheal durch den Isthmus miteinander verbunden ist. Die Schilddrüse wiegt bei Erwachsenen üblicherweise zwischen 18 und 30 g.

Funktionseinheit der Schilddrüse ist der Follikel. Er ist mit einschichtigen kubischen Epithelzellen, den Thyreozyten, ausgekleidet. Die polar ausgerichteten Zellen mit großem, rundlichem Zellkern sind an ihrer basolateralen Oberfläche den Kapillaren bzw. dem Blutstrom zugewandt und umschließen mit ihrer apikalen Membran das Follikellumen, in dem sich extrazellulär das Kolloid mit großen Mengen an Thyreoglobulin befindet. Dieses Glykoprotein stellt den Synthese- und Depotort der Schilddrüsenhormone dar. Die Form der Follikel passt sich den jeweiligen Funktionsphasen der Schilddrüse an und hängt von verschiedenen Faktoren (z. B. Umwelttemperatur, Lebensalter, Gravidität, Höhe des Spiegels an thyroideastimulierendem Hormon [TSH]) ab. Die C‑Zellen kommen in der gesamten Schilddrüse vor, weisen jedoch in den hinteren und oberen Abschnitten der Schilddrüsenlappen ihre höchste Dichte auf.

**Abb. 1:**
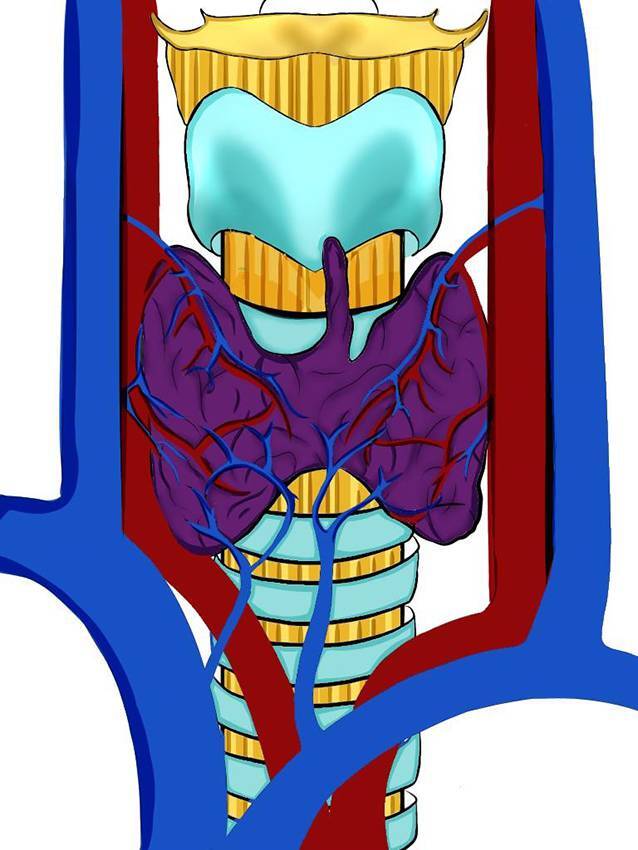
Schematische Darstellung der Schilddrüse, eigene Zeichnung

Embryologisch entsteht die Schilddrüse am Zungengrund aus dem Epithel des 2. Kiemenbogens. Vom Foramen caecum am Zungengrund wandert die Schilddrüse entlang des Ductus thyreoglossus nach distal und kommt etwa in der 7. Embryonalwoche an ihrer endgültigen Position an (Abb. 1). Der Ductus thyreoglossus verläuft teilweise anterior, teilweise posterior und selten durch das Zungenbein. Der Ductus thyreoglossus bildet sich üblicherweise zurück. Eventuelle Reste des Ductus thyreoglossus können sich später als mediane Halszysten oder mediane Halsfisteln manifestieren. Teilweise enthalten diese Reste von Schilddrüsengewebe, welches dann als ektopes Schilddrüsengewebe bezeichnet wird. Als Lobus pyramidalis wird ein embryonales Relikt des Ductus thyreoglossus bezeichnet, welcher zur Schilddrüse gehört und vom Isthmus nach oben zieht. Er soll einer aktuellen Metaanalyse zufolge in 42,8 % der Fälle vorhanden sein [1]. Theoretisch kann er bis zum Foramen caecum reichen. Er ist ein häufiger Grund für intraoperativ akzidentell zurückgelassenes Schilddrüsengewebe.

Die Schilddrüse ist eines der bestdurchbluteten Organe des menschlichen Körpers. Die A. thyroidea inferior entstammt üblicherweise dem Truncus thyreocervicalis und unterkreuzt die A. carotis communis am de-Quervain-Punkt (e1). Sie versorgt die unteren Anteile der Schilddrüse und zu 80 % die unteren Nebenschilddrüsen. Sie sollte daher schilddrüsennah ligiert werden. Das Gefäß tritt von laterokaudal an die Schilddrüse heran und teilt sich in unterschiedlicher Entfernung von der Drüse in mehrere Äste auf.

Die A. thyroidea superior entspringt als erster Ast der A. carotis externa und führt zum oberen Schilddrüsenpol. Die oberen Nebenschilddrüsen werden nur zu 20 % von dieser Arterie versorgt, sodass die Ligatur der A. thyreoidea superior weniger Probleme für die oberen Nebenschilddrüsen mit sich bringt (e2).

In 8 % der Fälle gibt es eine zusätzliche A. thyreoidea ima, welche direkt vom Aortenbogen zur Schilddrüse führt und vor der Trachea den Isthmus der Schilddrüse erreicht. Ist eine Ligatur der A. thyreoidea ima insuffizient, droht eine schwere intrathorakale Nachblutung (e1).

Der Schilddrüsenchirurg muss sich immer bewusst sein, dass die Gefäßverläufe sehr variabel sein können, was auch entsprechende Implikationen für den Verlauf das N. recurrens haben kann. So gibt es in selten Fällen einen direkten Zufluss aus der A. carotis interna und der Truncus brachiocephalicus kann sehr hoch stehen. Sollte sich die A. carotis communis rechts sehr nahe der Schilddrüse befinden, kann der N. laryngeus recurrens an dieser Stelle direkt vom N. vagus in die Schilddrüse ziehen, ohne den Umweg über das Mediastinum zu machen, sog. N. laryngeus recurrens non recurrens (e3).

Außerhalb der Organkapsel wird eine dünne Bindegewebsschicht, die sog. Grenzlamelle, beschrieben (e4). Zwischen beiden Schichten soll im Idealfall präpariert werden. Nach der persönlichen Erfahrung der Autoren lässt sich diese Schicht aber nicht immer identifizieren. Die Gefäße ziehen sowohl durch die Grenzlamelle als auch durch die Organkapsel und müssen daher stets drüsennah ligiert werden. Manchmal sitzt die Nebenschilddrüse direkt auf der Organkapsel und kann dann nicht erhalten werden. Es wird empfohlen, dieses Gewebe zu reimplantieren.

Chirurgisch relevante Nerven im Rahmen einer Schilddrüsenoperation sind die Nn. laryngei recurrentes und die Nn. laryngei superiores. Je nach Ausdehnung der Operation sind ggf. auch alle anderen Halsnerven betroffen. Diesbezüglich sei an dieser Stelle auf die einschlägige Literatur verwiesen.

Der N. laryngeus recurrens ist ein Ast des N. vagus und zieht rechts um den Truncus brachiocephalicus und links um den Aortenbogen. Durch letztere Tatsache können gelegentlich pulssynchrone Bewegungen des N. laryngeus recurrens links beobachtet werden, die bei dessen Auffindung helfen können. Der aszendierende Anteil des Nervs tritt hinter die Schilddrüse. Hier ist er besonders gefährdet. Insbesondere auf der rechten Seite gibt es eine Normvariante, im Sinne einer Ausstülpung des Schilddrüsenlappens nach posterior, das sog. Tuberculum Zuckerkandl. Hier liegt der Nerv der Schilddrüse häufig direkt an. Vor dem Eintritt des Nervs hinter den Ringknorpel kann er sich in bis zu 4 kleinere Äste aufteilen, von denen nur der vordere Ast in der Neurostimulation stimulierbar ist (e5). Auf den seltenen Fall eines N. laryngeus nonrecurrens rechts (0,6–0,8 %) wurde bereits hingewiesen. Die A. thyreoidea inferior eignet sich weniger zum Auffinden des Nervs, da Letzterer vor, hinter oder zwischen den Ästen der A. thyreoidea inferior verlaufen kann.

Die Lymphbahnen der Schilddrüse ziehen prälaryngeal, prä- und paratracheal zu den kaudalen Nn. cervicales profundi (e6).

## Physiologie

Die Schilddrüse produziert und speichert Schilddrüsenhormon. Dafür benötigt die Schilddrüse Jod. Der tägliche Jodbedarf von Erwachsenen wird mit 150–200 µg angegeben (e7). Die Bildung und Freisetzung der Schilddrüsenhormone unterliegt einem Regelkreis. Im Hypothalamus wird das Hormon TRH („thyreotropin releasing hormone“) gebildet und freigesetzt, wenn der Spiegel an Schilddrüsenhormonen (T3, T4) im Blut zu niedrig ist. TRH stimuliert in der Hypophyse die Ausschüttung von TSH (thyreoideastimulierendes Hormon). Die Freisetzung erfolgt pulsatil mit einer Halbwertszeit von 50–70 min. Sie unterliegt einer zirkadianen Rhythmik mit einem Maximum in der Nacht. TSH bindet an den G‑Protein-gekoppelten TSH-Rezeptor an der basolateralen Membran der Thyreozyten und führt somit in der Schilddrüse zur vermehrten Bildung von T3 und T4 sowie zu deren Freisetzung aus ihren Zwischenspeichern (Follikeln) ins Blut. Ein erhöhter T3- und T4-Spiegel im Blut hemmt die TRH- und TSH-Freisetzung im Sinne einer negativen Rückkopplung.

Schilddrüsenhormone sind im Blut zu 99 % an Plasmaproteine, v. a. Thyreoglobulin, gebunden und dadurch inaktiv. Thyreoglobulin wird ebenfalls in der Schilddrüse synthetisiert. Freies Schilddrüsenhormon (fT3 und fT4) erhöht den Grundumsatz, indem es die Herzarbeit, die Körpertemperatur sowie den Abbau von Fetten und Glykogen steigert. Im Einzelnen fördern Schilddrüsenhormone die Aufnahme von Glukose, den Kohlenhydratumsatz, den Sauerstoffverbrauch, die Wärmeproduktion, den Cholesterinabbau, die Entwicklung des zentralen Nervensystems, der Genitalorgane und des Knochenskeletts, die Muskelfunktion und erhöhen die Herzfrequenz und den systemischen Blutdruck. Gleichzeitig hemmen sie die Bildung energiereicher Phosphate, die Speicherung von Kohlenhydraten, die Bildung von Proteinen und die Energieausnutzung.

Für den Schilddrüsenchirurgen ist es essenziell zu wissen, dass die Schilddrüsenfollikel Schilddrüsenhormon speichern, welches bei der Manipulation im Rahmen einer Operation ins Blut mobilisiert werden kann. Daher ist präoperativ eine Hyperthyreose auszuschließen, damit es intraoperativ nicht zu einer thyreotoxischen Krise kommen kann.

In den C‑Zellen der Schilddrüse wird Calcitonin, ein Peptidhormon aus 32 Aminosäuren, synthetisiert. Es wirkt als spezifischer Antagonist von Parathormon und hemmt die Kalziumfreisetzung. Vermutlich liegt seine Hauptaufgabe in der Feinregulierung des Kalzium-Blutplasmaspiegels. Calcitonin bewirkt im Knochen eine Hemmung der Osteolyse. In den Nieren fördert es die Ausscheidung von Kalzium und Phosphat. Im Gastrointestinaltrakt verlangsamt Calcitonin die Verdauungsprozesse und damit die Resorption von Kalzium.

## Benigne Schilddrüsenerkrankungen

Benigne Erkrankungen der Schilddrüse gehören mit etwa 15 Mio. Betroffenen zu den häufigsten Erkrankungen in Deutschland. Sie manifestieren sich durch die von ihnen verursachten Funktionsstörungen (Schilddrüsenüber- oder -unterfunktion) oder durch die Folgen einer vergrößerten Schilddrüse mit Druck auf das umliegende Gewebe. Denkbare Symptome sind neben der ästhetischen Beeinträchtigung Schmerzen, Engegefühl, Luftnot, Schädigung des N. laryngeus recurrens oder eine obere Einflussstauung.

## Schilddrüsenfunktionsstörungen

### Hypothyreose

Die Schilddrüsenunterfunktion kann hereditär sein oder bildet sich bereits pränatal aus. In seltenen Fällen kann die Schilddrüse komplett fehlen (Schilddrüsenaplasie) oder zu klein für die Versorgung des Körpers mit den lebenswichtigen Schilddrüsenhormonen sein.

Andere häufige Ursachen sind die Hashimoto-Thyreoiditis, Jodmangelernährung, iatrogene Ursachen wie Schilddrüsenoperationen oder die Radiojodtherapie und Überdosierung von Thyreostatika.

Die Symptome der Hypothyreose können vielfältig sein, weshalb die Anamnese schwierig sein kann. Mögliche Symptome gemäß Deutschem Schilddrüsenzentrum (e8) sind: extreme Müdigkeit, übermäßig schnelle Erschöpfung, depressive Verstimmung, Konzentrationsstörungen, Antriebsmangel, Kopfschmerzen, Desinteresse, Kälteempfindlichkeit, Appetitlosigkeit, Verstopfung, erhöhte Infektanfälligkeit, kühle, trockene Haut, geschwollenes Gesicht, geschwollene Zunge und Augenpartien, stumpfe Haare, Haarausfall und Gewichtszunahme.

Die Therapie besteht in der Substitution von Schilddrüsenhormonen. Synthetische, also rein chemisch hergestellte Schilddrüsenhormone gibt es in verschiedenen Dosierungen als reine Thyroxin-Tabletten (T4), als reine T3-Tabletten und als Kombinationspräparate mit T4 und T3. Da der Körper je nach Bedarf aus dem langlebigeren und weniger wirksamen T4 durch die Abspaltung eines Jod-Atoms das stärker wirksamere und kurzlebigere T3 herstellen kann, favorisieren die meisten Experten wegen der besseren Steuerbarkeit und Verträglichkeit die Monotherapie mit synthetischem Thyroxin (T4). Trotz Thyroxin-Gabe und Normalisierung der Hormonspiegel leidet ein Teil der Patienten weiter unter typischen Symptomen einer Hypothyreose. Erst dann empfehlen Experten einen Therapieversuch mit T4-T3-Kombinationspräparaten (e8).

### Hyperthyreose

Die Hyperthyreose entspricht dem klinischen Symptomenkomplex, wenn der Körper in zu hohem Maße zirkulierenden Schilddrüsenhormonen ausgesetzt ist. Die Hyperthyreose wird über die freien Hormone fT3 und fT4 und das TSH im Serum bestimmt (e9).

Typische Symptome der Hyperthyreose sind Tachykardie, Hyperreflexie, Hitzeintoleranz, Schwitzen, Tremor, Nervosität, rasche Ermüdbarkeit, Gewichtsverlust, Appetitsteigerung, Schwäche, Diarrhöe und Dyspnoe. Ältere Patienten entwickeln weniger starke Symptome, aber häufiger kardiovaskuläre Komplikationen [2]. Eine Hyperthyreose erhöht die Gesamtmortalität, Herzinsuffizienz ist die Hauptursache (e10). Eine länger bestehende, unbehandelte Hyperthyreose erhöht die Inzidenz der Osteoporose (e11) und kann die Fruchtbarkeit herabsetzen (e12).

Die Tab. 1 fasst die klinischen Manifestationen der Hyperthyreose zusammen.

**Tab. 1:** Klinische Manifestation der Hyperthyreose nach de Leo et al. [3] (hier aus Maurer et al. [4])

Organsystem	Symptome	Klinische Zeichen
Konstitutionell	Gewichtsabnahme trotz gesteigerten Appetits, Temperaturstörungen wie Hitzeintoleranz, Schwitzen, Polydipsie	Gewichtsverlust
Kardiovaskulär	Palpitationen	Tachykardie, Hypertension, Vorhofflimmern
Pulmonal	Dyspnoe, Kurzatmigkeit	Tachypnoe
Gastrointestinal	Übelkeit, Erbrechen, häufige Defäkation	Abdominelle Beschwerden
Neuromuskulär	Tremor, Nervosität, Angststörung, Erschöpfung, Schwäche, gestörter Schlaf, Konzentrationsmangel	Extremitätentremor, Hyperaktivität, Hyperreflexie, Muskelschwäche in Becken und Rumpf
Dermal	Gesteigerte Perspiration	Warme, feuchte Haut
Reproduktion	–	Zyklusunregelmäßigkeiten
Ophthalmologisch (M. Basedow)	Diplopie, Augenreizung, Lidschwellung, retroorbitale Schmerzen	Proptose, Lidretraktion, verzögerter Lidschlag, periorbitales Ödem, Chemosis, Ophthalmoplegie

### Ursachen

Hyperthyreosen sind i. d. R. durch eine Überfunktion der Schilddrüse bedingt. Selten werden sie durch eine Überdosierung von Schilddrüsenhormon oder eine vermehrte Ausschüttung von Schilddrüsenhormon aus ektopen Quellen ausgelöst. Die häufigsten Formen der Hyperthyreose sind der M. Basedow, autonome Schilddrüsenknoten oder die disseminierte Autonomie. Die Prävalenz der Hyperthyreose wird mit 0,8 % in Europa angegeben [5]. Jodmangel soll eine leichte Hyperthyreose begünstigen (e13).

### M. Basedow

Das Krankheitsbild wurde in Deutschland erstmals 1840 von Carl von Basedow als eine Kombination aus Hyperthyreose, Exophthalmus und Struma (sog. Merseburger Trias) beschrieben und trägt im deutschsprachigen Raum den Namen des Erstbeschreibers. Im englischsprachigen Raum wird die Krankheit nach dem dortigen Erstbeschreiber „Graves’ disease“ genannt.

Die Autoimmunthyreopathie vom Typ Basedow ist die häufigste Form der Hyperthyreose mit einer Prävalenz von 0,5–1,5 % in der Bevölkerung (e14, [6]). Es handelt sich um eine Autoimmunerkrankung, bei der ein TSH-Rezeptor-Antikörper die Schilddrüse zu vermehrter Hormonausschüttung stimuliert. Dabei können isoliert T3 oder T4 oder beide betroffen sein. Es handelt sich also streng genommen nicht um eine Entzündung der Schilddrüse, sondern um eine autoimmun bedingte Überstimulation. Die Erkrankung kann in jedem Alter auftreten. Die höchste Prävalenz liegt im Alter zwischen 40 und 60 Jahren. Frauen sind 5‑mal häufiger betroffen. Ein relevanter Risikofaktor für die Entwicklung eines M. Basedow und einer endokrinen Orbitopathie ist Nikotinabusus (e15, e16). Außerdem werden Vitamin-D- und Selenmangel und immunmodulierende Medikamente diskutiert (e17).

Typisch ist der Nachweis von TSH-Rezeptor-Antikörpern (TRAK). Wenn eine endokrine Orbitopathie besteht, ist eine weiterführende Diagnostik prinzipiell nicht erforderlich. Bei erhöhten TRAK-Werten besteht in modernen Assays eine Sensitivität und Spezifität für das Vorliegen eines M. Basedow von über 90 % (e18). Hohe TPO-AK (Thyroxinperoxidase-Antikörper) sind eher typisch für eine Hashimoto-Thyreoiditis, kommen aber auch in etwa 70–80 % der Fälle beim M. Basedow und sogar bei Gesunden vor. Bei etwa der Hälfte der Basedow-Patienten findet auch eine Erhöhung der Thyreoglobulin-Antikörper.

In der Sonographie besteht meist eine Vergrößerung der Schilddrüse mit einer diffusen Echoarmut und Zeichen der Hypervaskularisierung in der Duplexsonographie. In der Schilddrüsenszintigraphie findet sich eine homogene und meist massiv erhöhte Tracerspeicherung in der Schilddrüse. Für die Diagnose eines M. Basedow ist eine Szintigraphie bei eindeutiger Befundlage allerdings entbehrlich (e19). Je nach Befundkombinationen können sich im Einzelfall sinnvolle Indikationen für weitere Untersuchungen ergeben (z. B. Nadelpunktionen bei sonographisch suspekten Knoten, Magnetresonanztomographie (MRT) der Augenhöhle bei V. a. eine endokrine Orbitopathie).

### Autonomie

Autonome Adenome und die disseminierte Schilddrüsenautonomie machen zusammen 50 % der Hyperthyreosen in Jodmangelgebieten aus (e20). Beide Erkrankungen können in jedem Alter auftreten, am häufigsten ab dem 40. Lebensjahr. Sie zeichnen sich durch das Fehlen von TSH-Autoantikörpern aus.

### Andere Ursachen

Durch Hypophysen- oder Hypothalamustumoren verursachte Hyperthyreosen werden als sekundäre bzw. tertiäre Hyperthyreosen bezeichnet. Eine Aufstellung weiterer Ursachen der Hyperthyreose findet sich bei Maurer und Holzer (Tab. 2; [4]).

**Tab. 2:** Ursachen der Hyperthyreose. (Aus Maurer et al. [4])

	Ätiologie	Diagnostik
*Häufige Ursachen*		
M. Basedow	TSH-R-AK binden an TSH-Rezeptor, TSH stimuliert die Schilddrüse	Vermehrter ^99m^Tc-Uptake mit diffuser Anreicherung in der Szintigraphie, positiver TSH-R-AK, ggf. erhöhte TPO-AK, ggf. Struma diffusa, ggf. eO, sonographisch echoarme Struma diffusa
Hyperfunktionelles Adenom	Autonom hormonproduzierender, gutartiger Schilddrüsentumor	Normaler oder erhöhter ^99m^Tc-Uptake mit lokalisierter Anreicherung in einem Knoten in der Szintigraphie, keine erhöhten Autoantikörper, sonographisch noduläre Anteile
Hyperthyreote Knotenstruma	Multiple autonom hormonproduzierende gutartige Schilddrüsentumoren	Normaler oder erhöhter ^99m^Tc-Uptake mit vielen fokalen Anreicherungen, keine erhöhten Autoantikörper, sonographisch multiple Nodi
Thyreotoxicosis factitia	Exogene Schilddrüsenhormonzufuhr	Geringer oder kein ^99m^Tc-Uptake, erniedrigte TPO
Schmerzlose, lymphozytäre Post-partum-Thyreoiditis	Autoimmune lymphozytäre Infiltration der Schilddrüse mit Freisetzung gespeicherter Schilddrüsenhormone	Auftreten innerhalb von 6 Monaten post partum
*Seltene Ursachen*		
Schmerzlose Thyreoiditis	Autoimmune lymphozytäre Infiltration der Schilddrüse mit Freisetzung gespeicherter Schilddrüsenhormone	Geringer oder kein ^99m^Tc-Uptake, TPO-AK niedrig oder nicht nachweisbar
Subakute Thyreoiditis	Thyreoiditis mit Freisetzung von gespeicherten Schilddrüsenhormonen, möglicherweise virale genese	Geringer oder kein ^99m^Tc-Uptake, TPO-AK nachweisbar
Chronische lymphozytäre Thyreoiditis (Hashimoto)	Autoimmune Stimulation und Produktion von B‑Zellen und Plasmazellen, die Antikörper gegen die Thyreozyten bilden	Im Anfangsstadium: Hyperthyreose („Hashitoxikose“), mit fortschreitender Zerstörung der Schilddrüse Entwicklung einer Hypothyreose. TPO-AK in 90 % positiv. ^99m^Tc-Uptake vermehrt. Sonographisch echoarme, inhomogene Struktur
Jodinduzierte Hyperthyreose	Exzessive Jodzufuhr	Geringer oder kein ^99m^Tc-Uptake
Medikamenteninduzierte Hyperthyreose (Lithium, Interferon-α)	Induktion einer autoimmunen Hyperthyreose oder inflammatorischen Thyreoiditis	Erhöhter ^99m^Tc-Uptake bei der autoimmunen Hyperthyreose. Erniedrigter oder kein ^99m^Tc-Uptake bei Thyreoiditis
Amiodaron-induzierte Thyreoiditis	Jodinduzierte Hyperthyreose (Typ 1), inflammatorische Thyreoiditis (Typ 2)	Erniedrigter oder kein ^99m^Tc-Uptake
*Sehr seltene Ursachen*		
TSH-produzierendes Hypophysenadenom	Hypophysenadenom	Erhöhtes TSH in Kombination mit erhöhten peripheren Schilddrüsenhormonen
Schwangerschaftshyperthyreose	Stimulation der Schilddrüsenhormonproduktion durch Choriongonadotropin	Schwangerschaft (**Cave**: Kontraindikation für Szintigraphie) in Kombination mit Hyperemesis oder Mehrlingsschwangerschaft
Molenschwangerschaft	Stimulation der Schilddrüsenhormonproduktion durch Choriongonadotropin	Molenschwangerschaft
Struma ovarii	Ovarialteratom mit Differenzierung in Schilddrüsenzellen	Geringer oder kein ^99m^Tc-Uptake in der Schilddrüse, dafür erhöhter ^99m^Tc-Uptake im kleinen Becken
Diffus metastasiertes follikuläres Schilddrüsenkarzinom	Schilddrüsenhormonproduktion durch ausgedehnte Tumormassen	Sichtbare Anreicherung im Ganzkörper-Scan

*TSH-R-AK* TSH-Rezeptor-Antikörper; *TSH* thyreoideastimulierendes Hormon, *TPO-AK* Thyreoperoxidase-Antikörper, *eO* endokrine Orbitopathie

### Endokrine Orbitopathie

Da beim M. Basedow auch außerhalb der Schilddrüse TSH-Rezeptoren exprimiert werden, gibt es zusätzlich extrathyreoidale Manifestationen, wobei die endokrine Orbitopathie (eO) hier die häufigste ist. Hier wird auf die einschlägige endokrinologische Literatur verwiesen. (e21).

### Thyreotoxische Krise

Die thyreotoxische Krise ist durch das Erreichen der individuellen Grenze zur Kompensation von Metabolismus, Thermoregulation und kardiovaskulärem System infolge einer Hyperthyreose definiert. Eine thyreotoxische Krise entwickelt sich bei weniger als 1 % der Hyperthyreosen.

Ursachlich können die Exposition mit jodhaltigem Röntgenkontrastmittel oder Amiodaron sein. Auch nach dem Absetzen einer thyreostatischen Medikation kann eine thyreotoxische Krise auftreten. Die Letalität wird mit 1,2–8 % angegeben (e22, [7]).

Die Therapie erfolgt unter intensivmedizinischen Bedingungen. Ziele der Behandlung sind die Wiederherstellung des Elektrolyt- und Flüssigkeitshaushalts, der Normothermie, die kardiorespiratorische Rekompensation und die Behandlung der zugrunde liegenden Pathologie.

### Therapie der Hyperthyreose

Grundsätzlich stehen 3 Therapiemöglichkeiten zur Verfügung, nämlich die medikamentöse Therapie mit Thyreostatika, die Radiojodtherapie und die Operation.

Thyreostatika sind entweder Thionamide, die über eine Hemmung der Thyreoperoxidase die Schilddrüsenhormonsynthese hemmen, oder Perchlorate, die die Aufnahme von Jod in die Thyreozyten blockieren. Beide Medikamentengruppen können über eine reaktive Erhöhung des TSH-Werts und den damit verbundenen Wachstumsreiz eine diffuse Struma bedingen. Perchlorate wirken schnell, verfügen aber über eine hohe Nebenwirkungsrate (Gastritis, Allergie, Lymphadenopathie) und werden v. a. protektiv in Situationen erhöhter Jodbelastung verabreicht. Die Thionamide Thiamazol und Carbimazol sind Medikamente der ersten Wahl bei nichtjodinduzierter Hyperthyreose. Bei Unverträglichkeit oder in der Schwangerschaft kann Propylthiouracil eingesetzt werden. Da der Wirkungseintritt von Thionamiden erst nach 6–8 Tagen eintritt, ist eine begleitende symptomatische Therapie der Hyperthyreose geboten. Thionamide sind potenziell hämatotoxisch, woraus eine Leukopenie bis hin zur Agranulozytose resultieren kann. Außerdem können Hepatotoxizität, Arthralgien und Hautreaktionen auftreten. Bei autonomen Adenomen oder hyperthyreoten Strumen sind Thyreostatika weniger indiziert, da eine Remission nicht zu erwarten ist (e23). Stattdessen werden Radiojodtherapie bzw. Operation bevorzugt. Thyreostatika werden präoperativ eingesetzt, um eine euthyreote Stoffwechsellage während der Operation zu erreichen. Dies gilt der Vermeidung intraoperativer, thyreotoxischer Krisen. Beim M. Basedow kann nach einjähriger thyreostatischer Therapie in bis zu 50–60 % der Fälle mit einer Remission gerechnet werden [4].

Die Radiojodtherapie besteht in der systemischen Applikation des β‑ und γ‑Strahlers Jod^131^ als Natriumjodid mit dem Ziel, hohe intrathyreoidale Strahlendosen zu erreichen. Ziele der Radiojodtherapie sind Beseitigung der Hyperthyreose, Beseitigung der Autonomie, Volumenreduktion der Struma, Antigenreduktion zwecks Verminderung der autoimmunogenen Basedow-Aktivität (e24). Gemäß Leitlinie der Deutschen Gesellschaft für Nuklearmedizin gelten folgende Indikationen: manifeste oder latente Hyperthyreose bei Autonomie oder M. Basedow (mit Indikation zur definitiven Therapie), Struma mit/ggf. ohne funktionelle Autonomie, Rezidivstruma mit und ohne funktionelle Autonomie (e25). Kontraindikationen sind Schwangerschaft und Stillzeit. Ein Zeitintervall von 4 Monaten bis zum Eintritt einer Schwangerschaft soll gewährleistet sein. Das Stillen sollte, wenn möglich, 3 Monate vor einer Radiojodtherapie beendet werden, um die Strahlenexposition der Mammae zu verringern (e26).

Zur Durchführung der Radiojodtherapie wird auf die Fachliteratur verwiesen (e25).

## Entzündungen

Etwa 20 % der Schilddrüsenerkrankungen sind entzündlicher Natur, wobei es sehr unterschiedliche Arten der Thyreoiditis gibt. Die meisten Formen sind autoimmun bedingt. Verbreitet und anerkannt ist die pathologische Einteilung nach Synoraki (Tab. 3; [8]).

**Tab. 3:** Thyreoiditisformen. (Aus [8] Synoraki S et al. Pathologe 2016; 37: 215–23)

A) autoimmunogene Schilddrüsenentzündungen	1. Autoimmunthyreoiditis (AIT) mit oder ohne subklinische/manifeste Hypothyreose	Hashimoto-Thyreoiditis (AIT mit Struma)
		Fibrosierende Variante der Hashimoto-Thyreoiditis (mit Struma)
		Fibrös-atrophische AIT (Ord-Thyreoiditis)
	2. Varianten autoimmunogener Schilddrüsenentzündungen	Post-partum-Thyreoiditis (PPT)
		Schmerzlose („silent“) Thyreoiditis
		Subakute Thyreoiditis de Quervain
B) nichtautoimmunogene Schilddrüsenentzündungen	1. akute infektiöse Schilddrüsenentzündungen	Bakterien
		Pilze
		Parasiten
		Viren
	2. Strahlen-bedingte Schilddrüsenentzündung	
C) invasiv-sklerosierende Perithyreoiditis Riedel („eisenharte Riedel-Struma“)		
D) Sonderformen der Schilddrüsenentzündungen	Palpationsthyreoiditis	
	Sarkoidose	
	Granulomatöse Polyangiitis (früher: M. Wegener)	
	Postoperativ nekrotisierende Thyreoiditis	
	Malakoplakie	
	Medikamenteninduzierte Thyreoiditis	

### Autoimmun bedingte Entzündungen

Die mit Abstand häufigste Form der Thyreoiditis ist die Autoimmunthyreoiditis (AIT) mit und ohne subklinische oder manifeste Hypothyreose. Mit dem M. Basedow wird die AIT unter dem Überbegriff „autoimmune thyroid disease“ zusammengefasst [9]. Die weniger häufigen Formen autoimmunogener Thyreoiditiden umfassen die subakute Thyreoiditis de Quervain, die Post-partum-Thyreoiditis und die spontane schmerzlose („silent“) Thyreoiditis. Die invasiv-sklerosierende (Peri‑)Thyreoiditis Riedel stellt eine Sonderform dar, die zwar sowohl morphologische als auch klinische Zeichen einer immunogenen Thyreoiditis zeigt, welche aber nur als sekundäre Folge eines primären Immunglobulin(IgG)4-assoziierten (multifokalen) sklerosierenden Prozesses anzusehen sind [8].

Bei bis zu 10 % der Menschen können Autoantikörper gegen Schilddrüsengewebe nachgewiesen werden, etwa 1 % entwickelt im Laufe des Lebens eine manifeste AIT [10, 11]. Die AIT stellt den Prototyp einer Autoimmunerkrankung dar, bei der es zum Verlust der während der Entwicklung des Organismus erworbenen Toleranz gegen Eigenantigene kommt, weshalb die auftretenden Autoantikörper nicht die Auslöser der Erkrankung, sondern deren Folge sind. Der für die Erkrankung relevante Prozess beginnt mit einer Aktivierung von gegen Schilddrüsenantigene gerichteten spezifischen T‑Helfer-Zellen [e27, e28], die einer bisher unbewiesenen Theorie nach durch eine virale Infektion ausgelöst wird [e29]; weitgehend gesichert erscheint jedoch aufgrund epidemiologischer Daten eine starke genetische Komponente in der Pathogenese der AIT [e30, e31].

#### Hashimoto-AIT

Bei der klassischen AIT Hashimoto (Erstbeschreibung 1912 von Hakaru Hashimoto) kommt es zur Ausbildung von Thyreoperoxidase-Antikörpern (TPO-AK), die im weit überwiegenden Teil der Patienten nachweisbar sind, und zur Ausbildung von Antikörpern gegen Thyreoglobulin (Tg-AK oder TAK). Letztere sind nur bei einem geringeren Anteil der Patienten signifikant erhöht. Sehr selten liegt eine Hashimoto-AIT auch ohne Antikörpernachweis vor. Die Diagnose wird dann anhand anderer Kriterien (Struma ohne andere Erklärung oder lymphozytäre Infiltration in der Schilddrüsenpunktion) gestellt. Die Erkrankung führt auf Dauer zu einer Schilddrüsenunterfunktion, wobei sich zu Beginn – bedingt durch die Zerstörung des Schilddrüsengewebes – auch Phasen der Überfunktion zeigen können. Dazwischen liegt häufig eine euthyreote Phase. Frauen sind mindestens 10-mal häufiger betroffen als Männer ([9, 11], e32, e33, e34).

Der Krankheitsverlauf ist bei den meisten Erkrankten leicht und bei einigen mittelschwer oder schwer. Die Symptome sind vielfältig und – gerade am Beginn der Erkrankung – schwierig einzuordnen. Die Hashimoto-Thyreoiditis ist derzeit nicht heilbar; sie kann nicht ursächlich behandelt werden. Wenn die Schilddrüse wegen ihrer chronischen Entzündung nicht mehr ausreichend Schilddrüsenhormone herstellen kann, muss die Unterfunktion durch eine Substitution therapiert werden. Bei symptomatischer Struma oder Schmerzen oder Malignomverdacht ist eine Thyreoidektomie angezeigt [12]. In extrem seltenen Fällen geht mit der Krankheit die Hashimoto-Enzephalopathie einher mit neurologischen Symptomen wie epileptischen Anfällen oder psychiatrischen Symptomen wie Halluzinationen.

Die Diagnostik umfasst neben den Laboruntersuchungen (Schilddrüsenhormonstatus und Antikörper) eine Ultraschalluntersuchung. Die Schilddrüse erscheint im Ultraschall inhomogen und echoarm. Zudem kann die in der Dopplersonographie erkennbare verstärkte Durchblutung ein Hinweis auf eine Entzündung sein. Die Größe der Schilddrüse ist wenig wegweisend, denn es gibt die klassische hypertrophe Form (Schilddrüsenvolumen > 25 ml) und eine atrophe Form (sog. Ord-Thyreoiditis). Histologisch (z. B. Grobnadelpunktion) ist das Follikelepithel äußerst variabel durch ein fokales bis weitgehend diffuses lymphoplasmazelluläres, polyklonales Infiltrat aus T‑Zellen mit unterschiedlich ausgeprägter Entwicklung von B‑Zell-haltigen Keimzentren destruiert [13]. Zur Unterscheidung zwischen Hashimoto-AIT und M. Basedow kann eine Schilddrüsenszintigraphie hilfreich sein. Der Uptake des Radiopharmakons ist bei der Hashimoto-AIT reduziert und beim M. Basedow erhöht.

Für die Diagnostik einer Hashimoto-Thyreoiditis wurden unterschiedliche Kriteriensysteme entwickelt, die inhaltlich ähnlich sind. Nach den Kriterien der Japan Thyroid Association wird folgendermaßen entschieden (Tab. 4; [14]):

- Definitive Diagnose: A + mindestens ein Kriterium aus B erfüllt
- Verdachtsdiagnose 1: Hypothyreose ohne andere erklärbare Ursache
- Verdachtsdiagnose 2: Antikörper ohne Schilddrüsenfunktionsstörung oder Struma
- Mögliche Diagnose: hypoechogene und/oder inhomogene Struktur in der Schilddrüsensonographie

**Tab. 4:** Kriteriensystem der Japan Thyroid Association für die Diagnosestellung einer Hashimoto-Thyreoiditis. (Nach 14)

Klasse	Kriterien
Klinische Zeichen (A)	Vergrößerte Schilddrüse (Struma) ohne andere erklärende Ursache (z. B. Jodmangel, M. Basedow oder Schilddrüsenautonomie)
Laborbefunde (B)	Positive MAK- bzw. TPO-Antikörper
	Positive Tg-Antikörper
	Lymphozytäre Infiltration in der punktionszytologischen Untersuchung

*MAK* mikrosomale Antikörper, *TPO* Thyroxinperoxidase

#### Post-partum-Thyreoiditis

Nach der Entbindung tritt bei etwa 5–8 % der Frauen eine sog. Post-partum-Thyreoiditis auf. Diese wird auf den Wegfall schwangerschaftsbedingter Suppression humoraler und zellulärer Immunreaktionen zurückgeführt. Prädisponierende Faktoren sollen Diabetes mellitus und/oder der Nachweis von Schilddrüsen-Autoantikörpern während der Schwangerschaft oder eine Hashimoto-AIT schon vor der Schwangerschaft sein (e35, e36). Bei wiederholter Schwangerschaft steigt das Risiko auf ein Rezidiv auf 70 % (e37).

Die Klinik ist variabel. Typischerweise tritt eine hyperthyreote Phase 4–8 Wochen post partum auf, die etwa 1–2 Monate anhält. Danach folgt eine vorübergehende hypothyreote Phase von 4–6 Monaten, bevor sich die Schilddrüse erholt. Nach spätestens 12 Monaten sind fast alle Patientinnen wieder euthyreot (e37). Allerdings gibt es in bis zu 50 % Verläufe, bei denen sich nach initialer Erholung eine dauerhafte Hypothyreose entwickelt.

Wegen der Kürze der hyperthyreoten Phase werden Thyreostatika nicht empfohlen. Bei Symptomen wie Herzklopfen oder Ängstlichkeit werden Betablocker empfohlen. Auf längere Sicht kann eine Substitution von Schilddrüsenhormonen nötig werden (e35).

#### Schmerzlose Thyreoiditis

Bei der schmerzlosen („silent“) Thyreoiditis handelt es sich um eine von einer Schwangerschaft unabhängige, aber klinisch ähnlich verlaufende Thyreoiditis. Morphologisch ähnelt sie der Post-partum-Thyreoiditis. Als Auslöser werden Viren (z. B. SARS-CoV-2) und Impfungen (COVID-19) beschrieben (e38, e39, e40).

Die Therapie richtet sich nach der Schilddrüsenhormonlage.

#### Subakute Thyreoiditis de Quervain

Diese Erkrankung hat viele Synonyme: subakute nichteitrige Thyreoiditis, granulomatöse, pseudotuberkulöse, pseudoriesenzellige oder riesenzellige Thyreoiditis, schleichende Thyreoiditis und Struma granulomatosa.

Die subakute Thyreoiditis de Quervain ist eine in Schüben verlaufende Erkrankung mit typischer Klinik und Morphologie. Sie ist die häufigste schmerzhafte Schilddrüsenentzündung und macht etwa 5 % der Schilddrüsenerkrankungen aus. Sie tritt bei Frauen 4‑ bis 7‑mal häufiger auf als bei Männern. Sie wird häufig nach viralen Infekten beobachtet.

Im Vordergrund der Beschwerden stehen ausgeprägte lokale Schmerzen in Hals‑, Ohr- und Kieferregion. Es können Schluckbeschwerden, Allgemeinsymptome wie Krankheitsgefühl und Gliederschmerzen auftreten. Die Schilddrüse ist typischerweise auf die 2‑ bis 3‑fache Größe vergrößert. Histologisch lassen sich unterschiedliche Phasen der Entzündung nachweisen, die aber auch nebeneinander vorkommen können. Durch die Zerstörung der Follikel in der frühen Phase tritt Kolloid aus. Es dominieren Mikroabszesse und neutrophile Granulozyten. In der floriden Phase dominieren Lymphozyten, Plasmazellen und Histiozyten. Es kommt zu einer Granulombildung. In der finalen Regenerationsphase sieht man eine lokale Fibrose [13].

Die Erkrankung verläuft i. d. R. binnen Wochen bis Monaten selbstlimitierend. Die über mehrere Monate andauernde Entzündung der Schilddrüse läuft typischerweise in mehreren Phasen ab. Zunächst kommt es zu einer Hyperthyreose, anschließend folgt eine kurz andauernde Euthyreose. Diese geht in eine Phase der Hypothyreose über. Während einer Erholungsphase normalisiert sich schließlich die Schilddrüsenfunktion wieder, und es kommt i. d. R. zu einer vollständigen Ausheilung.

Die Diagnose wird anhand der Anamnese, der klinischen Untersuchungen und mithilfe von Laborparametern gestellt. Im Zweifel kann mittels einer Gewebeentnahme (Biopsie) die Diagnose gesichert werden. Typisch ist eine druckschmerzhafte Schilddrüse, die asymmetrisch vergrößert sein kann. Im Ultraschall imponieren die schlecht abgrenzbaren entzündeten Areale echoarm. Die Vaskularisation ist vermindert, was die subakute Thyreoiditis de Quervain vom M. Basedow unterscheidet (e41). Im Labor zeigen sich ein erhöhtes C‑reaktives Protein (CRP), eine beschleunigte Blutsenkung und eine leichte Leukozytose (e42). Eine typische Antikörperkonstellation gibt es nicht. Die Bestimmung der Antikörper ist für die Differenzialdiagnose wichtig. Letztlich ist die Genese der subakuten Thyreoiditis de Quervain noch unklar.

Die Therapie erfolgt symptomatisch zunächst mit nichtsteroidalen Antirheumatika (NSAR). Wenn diese nicht ausreichend erfolgreich sind, werden Glukokortikoide, in erster Linie Prednisolon, eingesetzt. Hilft beides nicht, sollte die Diagnose hinterfragt werden (e43). Thyreostatika spielen keine Rolle, da die Schilddrüsenhormonproduktion bei der subakuten Thyreoiditis de Quervain nicht gesteigert ist. Sollten Symptome einer Hyperthyreose durch die Ausschüttung schon in der Schilddrüse vorhandenen Schilddrüsenhormons auftreten, wären diese symptomatisch zu behandeln. Sollte sich als Folgezustand eine Hypothyreose entwickeln, wäre diese zu substituieren.

### Nicht autoimmun bedingte Entzündungen

Ätiologisch werden akut-infektiöse und strahlenassoziierte Entzündungen unterschieden. Vorbestehende hyperplastische Knoten oder Neoplasien scheinen deren Entstehung zu begünstigen.

#### Infektiöse Thyreoiditis

Die akute eitrige Thyreoiditis ist selten (0,1–0,7 % der Schilddrüsenerkrankungen) und hat keine Alters- oder Geschlechtspräferenz. Die konservativ-antientzündliche Therapie richtet sich nach dem mikrobiologischen Erreger. Abszesse müssen ggf. chirurgisch entlastet werden. Bei Kindern sollte, insbesondere wenn die linke Seite betroffen ist, nach einer Sinus-piriformis-Fistel als seltene kongenitale Fehlbildung gesucht werden (e40, e44).

#### Strahlenassoziierte Thyreoiditis

Die strahlenassoziierte Thyreoiditis wird nach Radiojodtherapie bzw. perkutaner Bestrahlung beobachtet. Sie sind in ihrer Intensität abhängig vom Radioisotop und der Strahlendosis. Histologisch werden Follikelrupturen mit Nekrosen, Stromaödem, Infiltration durch segmentkernige Granulozyten beobachtet. Chronische Schäden weisen Fibrosen, aber auch eine chronische Thyreoiditis und Zellatypien auf.

Als strahlenbedingte Folgeerkrankungen gelten die Ausbildung von Hypothyreosen, multinodösen Strumen, Adenomen und Schilddrüsenkarzinomen. Nach perkutaner Strahlentherapie ist das strahlungsbedingte relative Risiko, ein Schilddrüsenmalignom zu entwickeln, gegenüber der Normalbevölkerung um das 15- bis 53-Fache erhöht. Für Radiojodtherapien ist ein solcher Zusammenhang bisher nicht belegt [13].

#### Invasiv-sklerosierende Perithyreoiditis

Die Riedel-Thyreoiditis, auch invasiv-sklerosierende (Peri‑)Thyreoiditis, eisenharte Struma Riedel, ist selten (0,06 % der Schilddrüsenerkrankungen). Frauen sind überwiegend betroffen. Es handelt sich um eine progrediente multifokale Fibrosklerose der Schilddrüse. Die Erkrankung schreitet i. d. R. bis zur kompletten Zerstörung des Organs fort. Dabei ist die Schilddrüse extrem hart und verursacht dadurch häufig Kompressionssymptome im Hals. Die Entzündung kann auf periglanduläres Gewebe übergreifen. Entsprechend sind Dyspnoe, Stridor, Heiserkeit und Dysphagie Symptome, die der Diagnose oft Monate vorausgehen.

Die Ätiologie ist noch nicht abschließend geklärt. Ein Zusammenhang mit IgG4-assoziierten Erkrankungen wird vielfach vermutet (e40).

Histologisch geht eine anfängliche Infiltration von überwiegend Lymphozyten und Plasmazellen mit Zerstörung der Schilddrüse und Beteiligung größerer Gefäße (Phlebitis) in einen fast vollständigen Ersatz des Schilddrüsenepithels in keloidähnliche Bindegewebszüge über, die als diagnostisches Charakteristikum auch im angrenzenden Bindegewebe nachweisbar sein müssen. Eine Differenzierung von Sarkomen, anaplastischen Schilddrüsenkarzinomen, diffus-sklerosierenden papillären Schilddrüsenkarzinomen und fibrosierenden Formen der Thyreoiditis muss erfolgen [13].

Zur Diagnostik gehören neben dem Schilddrüsenlabor auch die Abnahme von Entzündungsparametern (BSG oft leicht erhöht) und wegen eines oft begleitenden Hypoparathyreoidismus die Bestimmung des Kalziums und des Parathormons. In der ^18^Fluordesoxyglukose-Positronenemissionstomographie (^18^FDG-PET) lässt sich nicht nur die Aktivität der Entzündung in und außerhalb der Drüse bestimmen, es eignet sich auch zur Therapiekontrolle. Letztlich erfolgt die Diagnose histologisch über eine Stanzbiopsie oder im Rahmen einer dekompressiven Thyreoidektomie.

Eine spezifische Therapie steht nicht zur Verfügung. Die Chirurgie stellt den Therapiestandard zur Beseitigung der obstruktiven Symptome dar. Aufgrund des harten Gewebes und der periglandulären Entzündungen sind Komplikationen häufiger, sodass ggf. nur Isthmusresektionen durchgeführt werden sollten.

Außerdem steht die Substitutionstherapie von Schilddrüsenhormon und Kalzium im Vordergrund.

Konservativ antientzündliche Therapien sollen nach definitiver Diagnosestellung eingesetzt werden. Kortikosteroide wurden besonders bei aktiver Entzündung als effektiv beschrieben. Empfohlen werden Tagesdosen von 100 mg Prednisolon, wobei auch Dosen von 15–60 mg pro Tag über 3 Monate als erfolgreich beschrieben wurden. Im Fall des Scheiterns einer Kortikosteroidtherapie ist Tamoxifen das am häufigsten eingesetzte Medikament (e40).

#### Seltene Formen der Thyreoiditis

Die systemische Sarkoidose manifestiert sich in 1–4 % der Fälle auch in der Schilddrüse. Wenn eine Schilddrüsenfunktionsstörung dabei auftritt, ist sie i. d. R. gering ausgeprägt.

Auch eine systemische Amyloidose kann sich in der Schilddrüse manifestieren und zu einer Struma führen.

Da diese Erkrankungen sehr selten sind, wird an dieser Stelle auf die Fachliteratur verwiesen (e40).

## Ektopes Gewebe

### Ektopes Schilddrüsengewebe

Nicht vor oder lateral des dritten bis fünften Ringknorpels der Trachea und anatomisch getrennt von der Schilddrüse gelegenes funktionelles Schilddrüsengewebe wird per definitionem als ektopes oder akzessorisches Schilddrüsengewebe bezeichnet (e45). Ektopes Schilddrüsengewebe entsteht als entwicklungsgeschichtliche Abnormität, wenn im Rahmen des embryonalen Descensus Schilddrüsengewebe nicht in seiner finalen prätrachealen Lokalisation zu liegen kommt. Ektopes Schilddrüsengewebe findet sich im Verlauf des Ductus thyreoglossus oder kaudal der normalen Schilddrüsenlokalisation u. a. in Perikard, Herzmuskel, Vagina, der Leistenregion und der Leberpforte (e45). Die häufigste, und dem HNO-Arzt bekannte, relevante klinische Manifestation findet sich im Mundboden im Bereich des Foramen caecum. In 75 % dieser Fälle ist die linguale Schilddrüse das einzige nachweisbare ektope Schilddrüsengewebe, wobei meist eine angeborene Hypothyreose besteht (e46). Durch die Persistenz von Anteilen des Ductus thyreoglossus können (gelegentlich multiple) mediane Halszysten entstehen. In einem Prozent dieser Ductus-thyreoglossus-Zysten können Schilddrüsenmalignome entstehen. Meistens handelt es sich um papilläre Schilddrüsenkarzinome, andere Entitäten sind selten (e47, e48).

Selten kommen sog. parasitäre Schilddrüsenknoten vor, die keine Beziehung zur Schilddrüse oder Lymphknoten haben. Man geht von entwicklungsgeschichtlich abgetrenntem Schilddrüsengewebe aus (e49, e50, [13]), welches bei Jodmangel oder Schilddrüsenentzündungen durch Größenprogredienz symptomatisch werden kann.

### Ektopes Gewebe in der Schilddrüse

Etwa 20 % der Feten- und Neugeborenenschilddrüsen enthalten Thymusgewebe (e51). Dieses findet sich häufig nah der Schilddrüsenkapsel, kann selten aber auch tief in der Schilddrüse liegen. Auch Nebenschilddrüsengewebe und Epidermoidzysten werden gelegentlich bzw. selten in der Schilddrüse gefunden (e45, e52).

## Gutartige Schilddrüsentumoren

Das follikuläre Adenom ist eine benigne, gekapselte, nichtinvasive Neoplasie mit Zeichen der Follikelzelldifferenzierung, ohne dass die Kernkriterien des papillären Schilddrüsenkarzinoms erfüllt wären [15]. Adenome kommen in den unterschiedlichsten morphologischen Varianten vor, die keinerlei Einfluss auf das grundsätzlich benigne Verhalten dieser Tumoren haben. Die Differenzialdiagnose gegenüber dem follikulären Schilddrüsenkarzinom beruht auf dem ausschließlich histologisch zu führenden Ausschluss von Invasionszeichen (Angioinvasion, vollständiger Kapseldurchbruch). Insofern ist die Differenzialdiagnose zwischen einem follikulären Adenom und einem follikulären Schilddrüsenkarzinom i. d. R. weder zytologisch noch mit einer Grobnadelpunktion zu stellen. Onkozytär differenzierte Adenome werden nach der aktuellen Klassifikation gemäß Weltgesundheitsorganisation (WHO) als Hürthle-Zell-Adenome bezeichnet.

## Schilddrüsentumoren mit unsicherem/geringem Malignitätspotenzial

Die WHO unterscheidet neben benignen und malignen Tumoren auch solche Tumoren mit unsicherem bzw. geringem Malignitätspotenzial, nämlich

- den hyalisierenden trabekulären Tumor (HTT),
- den follikulären Tumor mit unsicherem Malignitätspotenzial (FT-UMP),
- den gut differenzierten Tumor mit unsicherem Malignitätspotenzial (DT-UMP),
- die nichtinvasive follikuläre Schilddrüsenneoplasie mit dem papillären Schilddrüsenkarzinom äquivalenten Kernmerkmalen.

Diesen Formen fehlen eindeutige Malignitätsmerkmale wie die Gefäßinvasion und der Kapseldurchbruch. Aus Kapazitätsgründen muss an dieser Stelle auf die Spezialliteratur verwiesen werden.

## Maligne Schilddrüsenerkrankungen

Mit einem Anteil von etwa 1 % zählt das Schilddrüsenkarzinom zu den seltenen Malignomen, stellt aber gleichzeitig die häufigste bösartige endokrine Neoplasie dar ([16], e53). Nach aktuellen Daten liegt die jährliche Inzidenzrate (WHO 2018) weltweit bei 10,2 (weiblich) bzw. 3,1 (männlich) und in Deutschland bei 11,1 bzw. 5,1 Neuerkrankungen pro 100.000 Einwohnern (e54). Die Gründe für die höhere Inzidenz bei Frauen sind bisher nicht geklärt. Das Erkrankungsalter liegt in Deutschland bei durchschnittlich 52 Jahren für Frauen und 56 Jahren für Männer (e54). In den letzten Jahren nimmt die weltweite Inzidenz der Schilddrüsenkarzinome zu. Das liegt aber allein an einer Zunahme der differenzierten papillären Schilddrüsenkarzinome (e55).

Malignome können aus allen in der Schilddrüse vorkommenden Zelltypen entstehen (Thyreozyten 80–85 %, C‑Zellen 5–10 %, aber auch Lymphozyten, Bestandteile des Stromas und des Gefäßsystems). Mehr als 99 % aller Schilddrüsenmalignome sind Karzinome, also epithelialer Herkunft [15, 17]. Diese lassen sich wiederum in follikeldifferenzierte, C‑Zell-differenzierte und seltene Schilddrüsenkarzinome (Tab. 5) unterteilen. Schilddrüsenkarzinome mit Follikelzelldifferenzierung werden nach WHO [15, 17] in 3 Gruppen eingeteilt, nämlich in differenzierte, in gering differenzierte und in anaplastische Schilddrüsenkarzinome. Die Prognose wird mit zunehmender Entdifferenzierung schlechter.

**Tab. 5:** Epitheliale Tumoren der Schilddrüse. (Aus Schmidt [13])

Maligne Schilddrüsenkarzinome
**1) Follikelzell-differenzierte Schilddrüsentumoren**
*a) differenzierte Karzinome*
i) papilläres Schilddrüsenkarzinom
ii) follikuläres Schilddrüsenkarzinom
iii) Hürthle-Zell-Karzinom
iv) differenziertes Karzinom NOS („not otherwise specified“)
**b) gering differenziertes Schilddrüsenkarzinom**
**c) anaplastisches Schilddrüsenkarzinom**
2) **C‑Zell-differenzierte Karzinome**
*a) medulläres Karzinom*
*b) gemischt medulläres und follikuläres Karzinom*
**1) Seltene Karzinome**
*a) Plattenepithelkarzinom*
*b) mukoepidermoides Karzinom*
*c) sklerosierendes mukoepidermoides Karzinom mit Eosinophilie*
*d) muzinöses Karzinom*
*e) Spindelzellkarzinom mit Thymusdifferenzierung (SETTLE)*
*f) intrathyreoidales Thymuskarzinom*

Außerdem gibt es nichtepitheliale Angiosarkome, Lymphommanifestationen und Metastasen in der Schilddrüse.

Im Rahmen dieser kurzen Übersicht kann nur auf die 4 häufigsten Entitäten, nämlich des papilläre Schilddrüsenkarzinom (PTC), das follikuläre Schilddrüsenkarzinom (FTC), das anaplastische Schilddrüsenkarzinom (ATC) und das medulläre Schilddrüsenkarzinom (MTC) eingegangen werden. Bezüglich der anderen Entitäten, die alle sehr selten auftreten (< 1 %), sei auf die Spezialliteratur hingewiesen ([13], e56).

Risikofaktoren für die Entstehung eines Schilddrüsenmalignoms sind Jodmangel und TSH-Erhöhung sowie Strahlenexposition und hier insbesondere Exposition gegenüber radioaktivem Jod. Auch genetische Veränderungen spielen eine große Rolle bei der Entstehung von Schilddrüsenmalignomen. Selten treten Schilddrüsentumoren im Rahmen einer multiplen endokrinen Metaplasie (MEN auf).

### Papilläres Schilddrüsenkarzinom

Mit etwa 60–70 % der Fälle ist das papilläre Schilddrüsenkarzinom (PTC) das häufigste Schilddrüsenkarzinom. Typisch ist eine Reihe distinktiver Kernmerkmale, die für die histologische Diagnosestellung ausschlaggebend sind. Das PTC bildet typischerweise papilläre Auswüchse, die ihm den Namen gegeben haben. Heute wird der Nachweis dieser Papillen neben dem Nachweis der Invasivität und zytologischer Merkmale zur Diagnosestellung gefordert, um das PTC von der nichtinvasiven follikulären Schilddrüsenneoplasie mit PTC-äquivalenten Kernmerkmalen (NIFTP) zu differenzieren. Auf molekularer Ebene werden 2 Typen unterschieden, nämlich eine BRAF-prädominante Signatur (2/3 der PTC; überwiegend BRAF-V600E-Mutatuion und seltener diverse Fusionsgene mit BRAF oder RET) oder eine RAS-prädominante Signatur (13 % der PTC; H/K/NRAS-Mutationen, EIF1AX-Mutationen und verschiedene seltene Mutationen des BRAF-Gens).

Die Metastasierung des papillären Schilddrüsenkarzinoms ist bevorzugt lymphogen. Hämatogene Metastasierung ist äußerst selten. Lymphogene Metastasen besiedeln i. d. R. die regionären Lymphknoten und treten klinisch oft vor dem eigentlichen Primärtumor in Erscheinung.

Papilläre Schilddrüsenkarzinome produzieren keine Schilddrüsenhormone, sie sind nonfunktionell. Bei der Szintigraphie erscheinen sie als kalte Knoten, da kein Jod^131^ angereichert wird. Dennoch sind die Zellen des papillären Schilddrüsenkarzinoms zur Jodaufnahme befähigt, was sie der Radiojodtherapie zugänglich macht.

Bei der Untersuchung des Patienten sind eine knotig vergrößerte Schilddrüse und evtl. vergrößerte Lymphknoten im Hals- und Nackenbereich auffällig. Lymphknotenmetastasen finden sich dabei meistens bei relativ jungen Patienten (30.–50. Lebensjahr). Frauen sind häufiger betroffen als Männer.

Bei kleinen papillären Karzinomen (Durchmesser < 15 mm, gute Abgrenzbarkeit) ist die Resektion des gleichseitigen Schilddrüsenlappens (Hemithyreoidektomie) mit Entfernung der regionalen Halslymphknoten ausreichend, bei größeren papillären Karzinomen ist eine totale Thyreoidektomie mit beiderseitiger Neck-Dissection indiziert.

Um alle jodspeichernden Zellen des Körpers zu eliminieren, schließt sich bei den größeren Tumoren nach der kompletten Thyreoidektomie meist eine Radiojodtherapie an. Im Anschluss muss L‑Thyroxin lebenslang substituiert werden: Die Dosis wird dabei relativ hoch gewählt, um den TSH-Spiegel (wichtigster Wachstumsreiz der Schilddrüse!) niedrig zu halten.

Die Prognose des papillären Schilddrüsenkarzinoms ist bei jungen Patienten sehr gut (10-Jahres-Überlebensrate > 90 %). Ein hohes Alter und das Vorliegen von Fernmetastasen sind prognostisch ungünstig.

### Follikuläres Schilddrüsenkarzinom

Das follikuläre Schilddrüsenkarzinom (FTC) macht etwa 10–30 % der Schilddrüsenmalignome aus. Es ist ein Schilddrüsenkarzinom, welches nicht von Follikelzellen ausgeht, die die Kernkriterien des PTC zeigen. Die Tumoren sind üblicherweise gekapselt und zeigen ein invasives Wachstum. Die aktuelle WHO-Klassifikation von 2017 unterscheidet verschiedene Typen des FTC. Das „minimal-invasive FTC“ ist immer gekapselt. Die Diagnose beruht auf einem oder mehreren vollständigen Kapseldurchbrüchen bei gleichzeitigem histologischem Ausschluss einer Angioinvasion. Die Prognose ist extrem gut, weshalb die Therapie auf ein Hemithyreoidektomie der betroffenen Seite beschränkt bleiben kann. Das „gekapselte angioinvasive FTC“ dagegen zeigt Gefäßeinbrüche und fakultativ auch Kapseldurchbrüche. Das „breit invasive FTC“ hat den biologisch ungünstigsten Verlauf. Da die Invasion in Lymphgefäße und auch Lymphknotenmetastasen beim FTC sehr selten vorkommen, sollte der Nachweis von Lymphknotenmetastasen beim FTC immer die Frage nach der korrekten Diagnose stellen lassen. Die häufigste Differenzialdiagnose wären follikuläre, solide und trabekuläre Varianten des PTC und die nichtinvasive follikuläre Schilddrüsenneoplasie mit PTC-äquivalenten Kernmerkmalen (NIFTP).

Die Prognose des follikulären Schilddrüsenkarzinoms variiert je nach Tumorstadium deutlich (10-Jahres-Überlebensrate 50–90 %).

### Anaplastisches Schilddrüsenkarzinom

Das anaplastische Schilddrüsenkarzinom (ATC) ist ein hochaggressives Schilddrüsenmalignom, bestehend aus undifferenzierten Schilddrüsenzellen. Eine Follikeldifferenzierung ist nicht mehr nachweisbar. Außer einer aggressiven Chirurgie gibt es keine verlässliche Therapieoption. Das ATC hat die schlechteste Prognose und verursacht mehr als 90 % der tumorassoziierten Todesfälle bei Schilddrüsenkrebs. Die mittlere Überlebenszeit der Betroffenen beträgt nach der Diagnosestellung nur rund 6 Monate. Eine bessere Prognose haben ATC, die innerhalb eines differenzierten Karzinoms auftreten oder rein intrathyreoidal liegen.

### C-Zell-differenzierte Schilddrüsenkarzinome

C‑Zell-differenzierte Schilddrüsenkarzinome (medulläre Schilddrüsenkarzinome, MTC) gehen von C‑Zellen aus und sind mit etwa 5 % eher selten. C‑Zellen sind spezialisierte Zellen der Schilddrüse, die das Hormon Calcitonin bilden. Diese C‑Zellen befinden sich i. d. R. innerhalb der follikulären Basalmembran zwischen bzw. unter den Follikelepithelzellen der Schilddrüse. An das Lumen der kolloidgefüllten Follikel reichen sie nicht heran. Im Vergleich zu den Follikelepithelzellen erscheinen C‑Zellen größer und heller, da sie zytoplasmareicher sind.

Karzinome, die von C‑Zellen ausgehen, bilden häufig Calcitonin, sodass es sich als Tumormarker eignet. Hohe Calcitoninspiegel können Diarrhöen auslösen (e57).

Grundsätzlich werden familiär bedingte MTC (etwa 30 %) von spontanen MTC (etwa 70 %) unterschieden. Erstere sind im Rahmen einer multiplen endokrinen Metaplasie (MEN Typ 2a und 2b) autosomal-dominant vererbt und entwickeln sich aus neoplastischen C‑Zell-Hyperplasien (CCH) durch eine Gain-of-Function-Keimbahnmutation des RET-Proonkogens. Das histologische Bild ist variabel. Im Rahmen von MEN 2a und 2b treten auch Phäochromozytome und andere Tumoren auf. Ein entsprechendes Screening bei Patienten mit MEN 2a oder 2b ist geboten.

Nach 10 Jahren leben noch etwa 50–70 % der Patienten mit einem MTC.

### Staging des Schilddrüsenkarzinoms

Die aktuelle 8. Auflage [15, 17] der TNM-Klassifikation gilt für papilläre (PTC), follikuläre (FTC), schlecht differenzierte Schilddrüsenkarzinome (PDTC), Hürthle-Zell-Karzinome und anaplastische Schilddrüsenkarzinome (ATC; Tab. 6). Aus dem TNM-Stadium wird zur Prognoseabschätzung (Rezidivrate, tumorspezifische Morbidität) folgende Stadieneinteilung gemäß Union Internationale Contre le Cancer (UICC) vorgeschlagen (Tab. 7). Beide Systeme dienen der initialen Risikobeurteilung und sind damit von entscheidender Bedeutung für das Ausmaß der Resektion und ein wesentlicher Eckpfeiler des weiteren therapeutischen Vorgehens.

**Tab. 6:** TNM-Klassifikation von Schilddrüsenmalignomen [15]

T‑Kategorie	Größe	Ausdehnung
*TX*	Tumor nicht beurteilbar	–
*T0*	Kein Tumor nachweisbar	
*T1a*	Tumor ≤ 1 cm	Auf die Schilddrüse begrenzt
*T1b*	Tumor > 1 cm, aber ≤ 2 cm	Auf die Schilddrüse begrenzt
*T2*	Tumor > 2 cm, aber ≤ 4 cm	Auf die Schilddrüse begrenzt
*T3a*	Tumor > 4 cm	Auf die Schilddrüse begrenzt
*T3b*	Tumor jeglicher Größe	Makroskopische extrathyreoidale Ausbreitung in die infrahyoidale Muskulatur
*T4a*	Tumor jeglicher Größe	Tumor in beliebiger Größe mit makroskopischer Invasion in subkutanes Fettgewebe, Larynx, Trachea, Ösophagus, N. laryngeus recurrens
*T4b*	Tumor jeglicher Größe	Makroskopische Invasion in prävertebrale Faszie, A. carotis oder mediastinale Gefäße
*Anmerkung*	Alle Kategorien können in solitäre Tumoren (s) und multifokale Tumoren (m) unterschieden werden, wobei der größte Tumor die Klassifikation bestimmt	
*N‑Kategorie*	N‑Kriterium	
*NX*	Regionale Lymphknoten nicht beurteilbar	
*N0*	Keine regionalen Lymphknotenmetastasen nachweisbar	
*N0a*	Ein oder mehr zytologisch oder histologisch als gutartig bestätigte Lymphknoten	
*N0b*	Kein klinischer oder radiologischer Hinweis auf regionale Lymphknotenmetastasen	
*N1*	Regionale Lymphknotenmetastasen nachweisbar	
*N1a*	Metastasen in Level VI oder VII: prätracheal, paratracheal, prälaryngeal (Delphi-Lymphknoten oder oberes Mediastinum, kann ein- oder beidseitig sein)	
*N1b*	Unilaterale, bilaterale oder kontralaterale Metastasen (in Level I, II, III, IV oder V) oder retropharyngeale Lymphknotenmetastasen	
*M‑Kategorie*	M‑Kriterium	
*M0*	Keine Fernmetastasen	
*M1*	Nachweis von Fernmetastase(n)

**Tab. 7:** UICC-System zur Stadieneinteilung von Schilddrüsenkarzinomen [15]

**PTC und FTC, Alter <** **55 Jahre**			
*Stadium I*	Jedes T	Jedes N	M0
*Stadium II*	Jedes T	Jedes N	M1
**PTC und FTC, Alter ≥** **55 Jahre**			
*Stadium I*	T1a, T1b, T2	N0	M0
*Stadium II*	T3	N0	M0
	T1, T2, T3	N1	M0
*Stadium III*	T4a	Jedes N	M0
*Stadium IVa*	T4b	Jedes N	M0
*Stadium IVb*	Jedes T	Jedes N	M1
**MTC**			
*Stadium I*	T1a, T1b	N0	M0
*Stadium II*	T2, T3	N0	M0
*Stadium III*	T1, T2, T3	N1a	M0
*Stadium IVa*	T1, T2, T3	N1b	M0
	T4a	jedes N	M0
*Stadium IVb*	T4b	Jedes N	M0
*Stadium IVc*	Jedes T	Jedes N	M1
**ATC**			
*Stadium IVa*	T1, T2, T3a	N0	M0
*Stadium IVb*	T1, T2, T3a	N1	M0
	T3b, T4a, T4b	N0, N1	M0
*Stadium IVc*	Jedes T	Jedes N	M1

*ATC* anaplastisches Schilddrüsenkarzinom, *FTC* follikuläres Schilddrüsenkarzinom, *MTC* medulläres Schilddrüsenkarzinom, *PTC* papilläres Schilddrüsenkarzinom, *UICC *Union Internationale Contre le Cancer

## Schilddrüsenchirurgie

Wie in fast allen Bereichen der modernen Chirurgie richtet sich auch die moderne Schilddrüsenchirurgie nach dem Prinzip eines möglichst großen Funktionserhalts und einer möglichst geringen Morbidität. Das bedeutet, dass die noch vor wenigen Jahrzehnten großzügige Indikation zur totalen Thyreoidektomie heute nur noch selten gestellt wird [18]. Entsprechend werden je nach präoperativer Diagnose und präoperativem Befund die Enukleation eines Schilddrüsenknotens entlang seiner Kapsel, die Knotenexzision mit umgebendem Gewebesaum, die Isthmusresektion, die subtotale Lappenresektion (verbleibender Schilddrüsenrest < 4 g), die fast totale Lappenresektion (verbleibender Schilddrüsenrest < 1 g) und die komplette Hemithyreoidektomie unterschieden. Da sich intraoperativ auch eine Enukleation zu einer Lobektomie ausweiten kann, ist immer zu fordern, dass der Operateur über die notwendigen Kenntnisse und Fertigkeiten und die Klinik über die notwendigen Materialien und Hilfsmittel verfügt.

### Indikation zur Operation

Gemäß aktueller AWMF-S2k-Leitlinie [19] vom 05.12.2021 wird die Indikation zur operativen Behandlung auf der Basis der Erkrankungs- und Lokalisationsdiagnostik unter Abwägung der individuellen nichtoperativen Behandlungsverfahren und der möglichen Komplikationen einer operativen Behandlung gestellt. Ziel der operativen Behandlung ist die sichere und dauerhafte Beseitigung der zugrunde liegenden Schilddrüsenerkrankung. Gemäß Leitlinie

- soll eine präoperative Laryngoskopie zur Beurteilung der Stimmbandfunktion erfolgen.
- soll präoperativ das Serumkalzium bestimmt werden. Auffälligkeiten bedürfen der weiteren präoperativen Abklärung.
- sollte präoperativ das basale Calcitonin als möglicher Hinweis auf ein medulläres Schilddrüsenkarzinom bestimmt werden.
- sollte bei bildgebend suspekten Knoten > 1 cm nach Ausschluss einer Autonomie und bei nicht erhöhtem Calcitonin, wenn suspekte Halslymphknoten vorliegen oder der V. a. ein lokal invasives Wachstum besteht und wenn die zytologische Diagnose für die Operationsplanung von Bedeutung ist, eine Feinnadelaspirationszytologie erfolgen.

**Abb. 2:**
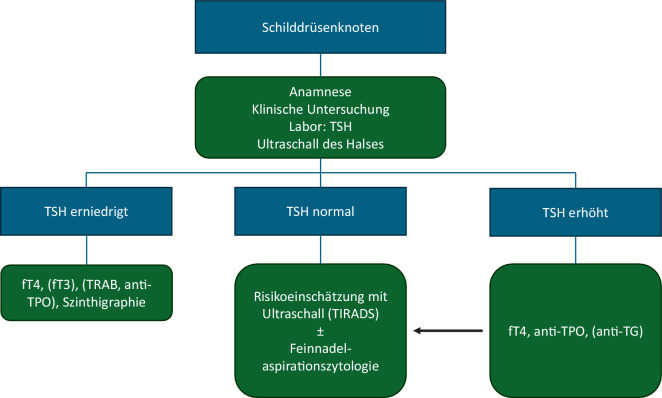
Vorgehen bei Schilddrüsenknoten gemäß European Thyroid Association 2023 [20]. *fT3* freies Trijodthyronin, *fT4* freies Thyroxin, *TG* Thyreoglobulin, *TIRADS *Thyroid Imaging Reporting And Data System, *TRAB* TSH-Rezeptor-Antikörper, *TPO* Thyreoperoxidase, *TSH *thyroideastimulierendes Hormon

In Kenntnis dieser Informationen empfahl die European Thyroid Association 2023 das in Abb. 2 dargestellte Vorgehen [20].

**Abb. 3:**
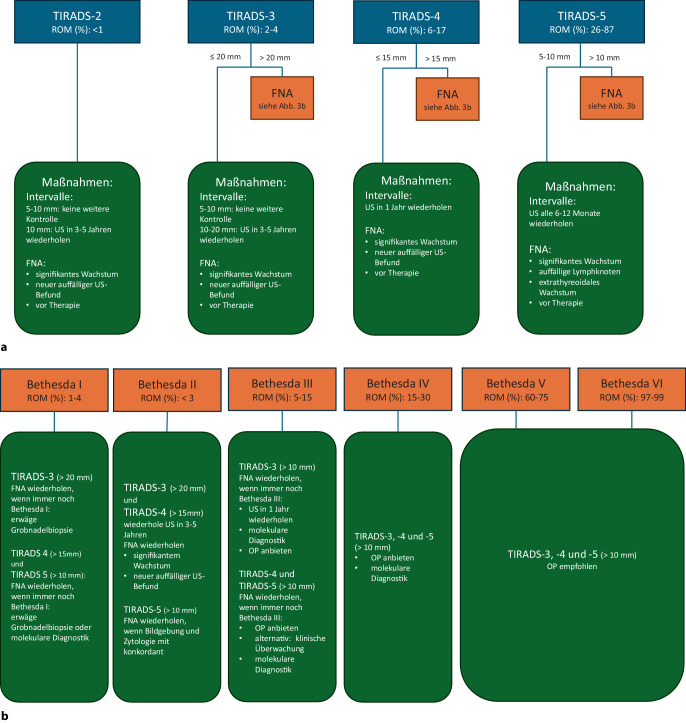
**a**, **b** Weiteres diagnostisches und therapeutisches Vorgehen je nach Ultraschallbefund gemäß European Thyroid Association 2023 [20]. **a** First Line Approach: Mache einen Ultraschall und bestimme das Malignitätsrisiko (ROM) gemäß der Europäischen TIRADS-Klassifikation. **b **Second Line Approach: Mache eine Feinnadelaspirationszytologie (FNA). *FNA* Feinnadelaspirationszytologie, *ROM* Malignitätsrisiko, *TIRADS *Thyroid Imaging Reporting And Data System, *US* Ultraschall

Die Ultraschalluntersuchung des Halsbereichs gehört gemäß Leitlinie zur Basisdiagnostik der Schilddrüsenerkrankungen [21]. Sie gibt Auskunft über Ausdehnung, Struktur des Organs, insbesondere bei knotigen Veränderungen Malignitätshinweise und extrathyreoidale pathologische Veränderungen, z. B. Lymphknotenvergrößerungen [e58]. Ein qualifizierter Ultraschall beinhaltet die Einordnung des Knotens in etablierte standardisierte Scoring-Systeme wie z. B. das TIRADS (Thyroid Imaging Reporting And Data System), von dem es u. a. eine amerikanische und eine europäische Version gibt [22, 23]. Verschiedene sonographische Parameter werden dabei in einem Punktesystem quantifiziert und ergeben einen von 5 TIRADS-Leveln, denen wiederum weitere therapeutische Empfehlungen zugeordnet sind (Abb. 3 aus [20]). Bei TIRADS-1-Knoten werden keine weiteren Maßnahmen oder Verlaufskontrollen empfohlen.

Die S2k-Leitlinie beschreibt die Szintigraphie nur noch als „kann erwogen werden“ und zwar vor Rezidiveingriffen, bei erniedrigtem TSH und bei V. a. retrosternmale/ektope Schilddrüsenanteile.

Zur Operationsplanung wird eine zusätzliche Bildgebung mittels MRT oder Computertomographie (CT) *ohne Kontrastmittel* empfohlen.

**Abb. 4:**
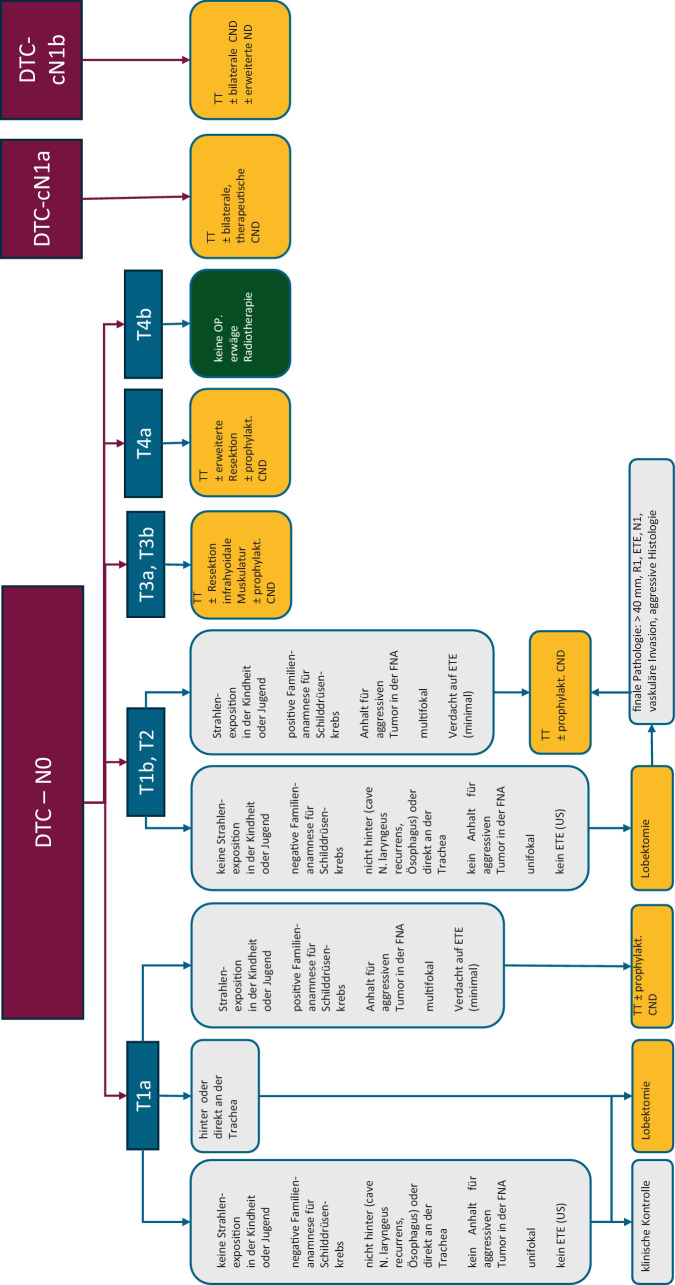
Chirurgisches Vorgehen bei differenzierten Schilddrüsenkarzinomen. (ESMO-Leitlinie 2019 [24]). *CND* zentrale Neck-Dissection (Regio 6), *CT* Computertomographie, *DTC* differenzierte Schilddrüsenkarzinome, *EBRT* Radiotherapie, *ESMO* European Society for Medical Oncology, *ETE* extrathyreoidale Tumorausbreitung, *LND* laterale Neck-Dissection, *MRI* Magnetresonanztomographie, *ND* Neck-Dissection, *TT* totale Thyreoidektomie, *US* Ultraschall)

Die aktuelle Leitlinie der European Society for Medical Oncology (ESMO) von 2019 [24] empfiehlt nachstehenden Algorithmus für das chirurgische Management von differenzierten Schilddrüsenkarzinomen (Abb. 4). Das Vorgehen ist in einigen Punkten im Vergleich zur Vorgängerleitlinie deeskaliert worden.

### Hilfsmittel

#### Neuromonitoring

Ein Neuromonitoring gilt heute national und international als Standard (e59, [25]). Die Auswertung der Schilddrüsendatenbank der Deutschen Gesellschaft für Allgemein- und Viszeralchirurgie ergab, dass 2019 schon 98 % der Schilddrüsenoperationen unter Neuromonitoring erfolgten [18].

Dem HNO-Arzt sind die Grundlagen des Neuromonitoring aus der Ohr- und Speicheldrüsenchirurgie hinreichend bekannt, weshalb an dieser Stelle auf die entsprechende Literatur verwiesen werden soll. Selbstverständlich darf der Patient allenfalls zur Narkoseeinleitung kurzzeitig relaxiert werden.

Für die Schilddrüse werden intermittierende von kontinuierlichen Stimulationssystemen unterschieden. Letztere sollen bei anspruchsvolleren Operationen, wie etwa Revisionsoperationen oder Eingriffen bei Malignomen Vorteile haben (e60, e61, e62). Die Stimulation erfolgt über den N. vagus oder seinen Endast, den N. laryngeus recurrens (NLR). Erfolgsorgan ist der M. vocalis.

Moderne Systeme verwenden auf den Intubationstubus aufzuklebende Oberflächenelektroden, die im Idealfall so angebracht werden, dass sie nach der Intubation der Stimmlippe anliegen. Der Vorteil liegt im nichtinvasiven Setting, der Nachteil darin, dass die Elektrodenlage nicht bzw. nur über eine Mikrolaryngoskopie kontrolliert werden kann. Auch Dislokationen des Tubus während der Schilddrüsen-Op. sind mögliche Fehlerquellen. Außerdem sind Klebeelektroden für Kindertuben nicht verfügbar. Alternativ können intraoperativ nach der Intubation Nadelelektroden über eine Mikrolaryngoskopie endolaryngeal in die Stimmlippen eingebracht werden. Eine dritte Möglichkeit besteht im Einbringen von Nadelelektroden über die Op.-Wunde durch die Membrana cricothyreoidea in den M. vocalis. Wir bevorzugen die auf den Tubus aufgeklebten Elektroden und steigen bei unzureichender Ableitung auf Nadelelektroden um, die wir steril über den operativen Situs durch die Membrana cricothyreoidea in den M. vocalis einbringen.

Für die intermittierende Reizung der jeweiligen Nerven stehen mono- und bipolare Reizsonden zur Verfügung. Da bipolare Sonden nur einen Teilbereich des Nervs stimulieren und außerdem unhandlicher sind, bevorzugen wir monopolare Reizelektroden. Empfohlen werden Reizstärken zwischen 1 und 2 mA (e63). Wir verwenden initial geringere Reizstärken (0,7 mA). Es gibt einen Vorschlag für standardisiertes Vorgehen der International Neuro Monitoring Study Group (Tab. 8; [26]). Diesem Vorschlag haben wir uns angeschlossen, um vorbestehende Stimmbandstillstände zu detektieren und weil auch bei klinisch komplettem Stimmbandstillstand manchmal ableitbare Potenziale messbar sind (e64).

**Tab. 8:** Vorschlag für standardisiertes Vorgehen der International Neuro Monitoring Study Group. (Nach Randolph et al. [26] )

L1	Präoperative Laryngoskopie zur Beurteilung der Intaktheit der Stimmbandfunktion
V1	Intaktes Stimulations-EMG des N. vagus vor der Dissektion des gleichseitigen Schilddrüsenlappens
R1	Intaktes Stimulations-EMG des NLR vor der Dissektion des gleichseitigen Schilddrüsenlappens
R2	Intaktes Stimulations-EMG des NLR nach der Dissektion des gleichseitigen Schilddrüsenlappens
V2	Intaktes Stimulations-EMG des N. vagus nach der Dissektion des gleichseitigen Schilddrüsenlappens
L2	Postoperative Laryngoskopie zur Beurteilung der Intaktheit der Stimmbandfunktion

*EMG *Elektromyogramm, *NLR* N. laryngeus recurrens

Die Darstellung des N. vagus ist dem HNO-Chirurgen geläufig. Wir schlingen den N. vagus gern mit einem Vessel-Loop an, um ihn zügig für die Nervenreizungen wiederzufinden. Ein Kilian-Spekulum ist hierfür zusätzlich sehr hilfreich, weil es die Exposition mit einer Hand erlaubt.

Für das kontinuierliche intraoperative Neuromonitoring wird eine APS^R^-Elektrode („automatic periodic stimulation“) um den N. vagus gelegt und mit einer Naht fixiert. Die Stimulation am NLR entfällt. Das System liefert optisch und akustisch kontinuierliche Informationen über die Nervenfunktion. Eine Schädigung fällt also sofort auf und kann eventuell eine weitere Schädigung verhindern.

Das Neuromonitoring bietet zusätzlich die Option der Funktionsüberprüfung des Ramus externus des N. laryngeus superior, der den M. cricothyreoideus innerviert. Seine Schädigung führt zu einem Ausfall der Vorspannung des Kehlkopfs, welche bei professionellen Sängern und Sprechern durchaus zu relevanten Stimmproblemen führen kann. Der Nerv ist insbesondere bei der Präparation des oberen Schilddrüsenpols in Gefahr.

**Abb. 5:**
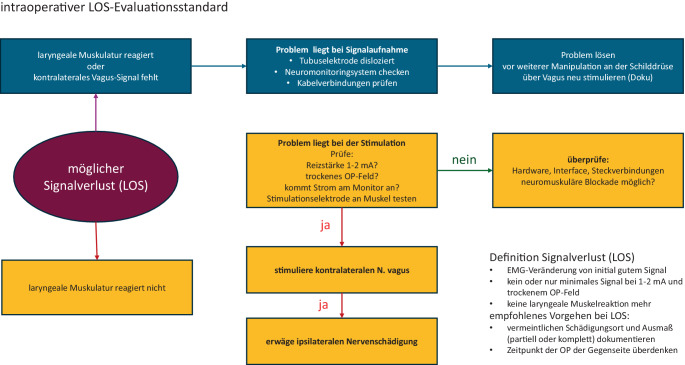
Algorithmus bei intraoperativem Verlust des Neuromonitoringsignals. (Aus [26].) *LOS* loss of signal, *ETT* Endotrachealtubus, *EMG* Elektromyogramm

Nach unserer Kenntnis konnte bisher nicht gezeigt werden, dass die Verwendung eines Neuromonitorings die Schädigung des NLR verhindern kann [25]. Trotzdem bietet das Neuromonitoring Vorteile gegenüber der rein visuellen Kontrolle des Nervs. Der wesentliche Vorteil besteht darin, dass ein Nervenschaden bereits intraoperativ entdeckt und eine eventuell einzeitig geplante Operation der Gegenseite zurückgestellt werden kann, bis klar ist, ob sich der Nerv funktionell erholt. In der chirurgischen Literatur gibt es dafür entsprechende Algorithmen (z. B. Abb. 5).

#### Versiegelnde Instrumente

Aufgrund der starken Gefäßversorgung der Schilddrüse sind zahlreiche Gefäßligaturen erforderlich. Konventionelle elektrochirurgische Instrumente erzeugen Hitze von 150–400 °C. Hierdurch können thermische Läsionen an Nerven und anderen Geweben entstehen. Daher wurden gefäßversiegelnde Hilfsmittel in die Schilddrüsenchirurgie eingeführt, die zwischenzeitlich zum Standardverfahren gehören ([27], e65, e66, e67).

Im Wesentlichen gibt es 2 Geräte. Das Harmonic Scalpel^R^ (Fa. Ethicon, Somerville/NJ, USA) ist eine ultraschallaktivierte Gerinnungsschere mit Ultraschallwellen bei einer Frequenz von 55 kHz. Es versiegelt die Gefäße durch eine Proteindenaturierung bei 50–100 °C. Die Vibration der Klinge bewirkt gleichzeitiges Schneiden und Koagulieren von Blutgefäßen, wobei etwas perivasales Bindegewebe hilfreich ist. Da nur eine Schneide heiß wird, lässt sich der periläsionale thermische Schaden durch geschickte Handhabung des Harmonic Scalpel^R^ weiter minimieren (e66, e68).

Das Liga-Sure^R^ (Fa. Medtronic, Minneapolis/MN, USA) arbeitet mit bipolarem, hochfrequentem Strom. Dabei resultiert die Abgabe von kontrollierter Energie zusammen mit einem physikalischen Druck in einer Versieglung der Gefäßwände ([27], e65, e67). Zum chirurgischen Vorgehen sei auf die Arbeit von O’Neill et al. verwiesen (e69).

Die wesentlichen Vorteile der Verwendung moderner versiegelnder Instrumente bestehen in der Reduktion der Op.-Zeit und der Reduktion des postoperativen Drainagevolumens. Eine eigene Diplomarbeit zum Thema (e70) ermittelte 20 Arbeiten mit insgesamt 6605 Schilddrüsenoperationen, die zwischen konventioneller (*n* = 1640), kombinierter (*n* = 60) und versiegelnder Technik (*n* = 4905) verglichen hatten. Unabhängig von der Art der Schilddrüsen-Op. wird eine durchschnittliche Reduktion der Op.-Zeiten um etwa 30 % beschrieben (101,5 vs. 71,4 min). Das Drainagevolumen lag bei durchschnittlich 130 ml bei konventioneller und bei 58 ml mit versiegelnder Op.-Technik. In unserem Review konnten wir keine signifikanten Unterschiede bezüglich der Komplikationen Infektion, Nachblutung, Hypokalzämien und Inzidenz von Rekurrensschäden zwischen den beiden versiegelnden Hilfsmitteln feststellen.

Zur Rate an Rekurrensparesen nimmt eine aktuelle Metaanalyse Stellung (e71). Es handelt sich um eine Zusammenstellung und Analyse von 40 Arbeiten mit 17.953 Patienten. Auch diese Arbeit beschreibt keine signifikanten Unterschiede zwischen versiegelnder Op.-Technik im Vergleich zur konventionellen Blutstillung.

Es gibt inzwischen mehrere Arbeiten, die trotz der zusätzlichen Kosten für die versiegelnden Instrumente infolge der verkürzten Op.-Zeiten zu einer positiven wirtschaftlichen Betrachtung kommen. Insofern sind versiegelnde Verfahren im Klinikum der beiden Autoren zum Standard geworden. Inwieweit der zunehmende ökonomische Druck und die Ambulantisierung hierauf Einfluss haben, bleibt zukünftig abzuwarten.

#### Autofluoreszenz und ICG-Fluoreszenz

Die Techniken der Autofluoreszenz und der Indozyaningrün(ICG)-Fluoreszenz können für die intraoperative Überprüfung der Durchblutung der Nebenschilddrüsen eingesetzt werden. Die Verfahren sind noch in der Entwicklung, die Machbarkeit konnte aber bereits gezeigt werden (e72). Die Autoren haben bisher keine eignen Erfahrungen mit der Technik.

### Operationstechnik

#### Zugang und Durchtrennung des Isthmus

Schilddrüsenresektionen erfolgen unter Vollnarkose. In der Regel verwenden wir auf den Tubus aufgeklebte Elektroden für das Neuromonitoring. Der Patient liegt in Rückenlage mit möglichst überstrecktem Kopf. Standard ist ein zur Mitte symmetrischer Kocher-Kragenschnitt. Die Länge bemisst sich nach der Größe der zu resezierenden Schilddrüse. Nach Durchtrennen von Haut, Subkutis und Platysma wird die Linea alba dargestellt und in sagittaler Richtung eröffnet. Je nach Größe der zu resezierenden Schilddrüse sollte vorher ein Haut-Platysma-Lappen gehoben werden. Danach erfolgt das Abheben der geraden Halsmuskulatur von der Schilddrüse und das Aufsuchen des Oberrands des Isthmus. Hier finden sich einige leicht zu verletzende Venen. Die Lage von Krikoid und Schildknorpel sollte überprüft werden, um im Weiteren eine akzidentelle Verletzung der Membrana cricothyreoidea zu vermeiden. Jetzt wird der Isthmus dargestellt und im Idealfall an dessen Unterrand die Trachea identifiziert. Der Isthmus wird jetzt unterfahren und durchtrennt. Wir verwenden dafür heute versiegelnde Hilfsmittel (z. B. UltraCision Harmonic Scalpel, Ethicon Deutschland, Norderstedt). Eine Naht der Schilddrüsenlappen ist in den meisten Fällen entbehrlich. Anschließend lösen wir die Schilddrüse vom vorderen Drittel der Tracheazirkumferenz ab. Dadurch wird der Schilddrüsenlappen für die spätere Präparation mobiler und der Situs übersichtlicher. Wenn streng auf der Trachea präpariert wird, ist eine Verletzung des N. laryngeus recurrens nicht zu erwarten.

#### Auslösen des oberen Schilddrüsenpols

Vor dem Absetzten des oberen Pols erfolgt die Freilegung der Hals-Gefäß-Nerven-Scheide. An typischer Stelle zwischen V. jugularis interna und A. carotis communis findet sich der N. vagus. Diesen schlingen wir grundsätzlich mit einem Vessel-Loop an, um ihn im Verlauf der Operation für eventuelle Stimulationen schnell wiederzufinden. Der Nerv wird stimuliert und die evozieren Kurven dokumentiert. Hier kann zwischen einer diskontinuierlichen Stimulation und einer kontinuierlichen gewählt werden. Sollten die abgeleiteten Kurven über den N. vagus nicht ausreichend sein, kann das auf eine Fehllage der Tubuselektroden hinweisen. Gelingt eine Replatzierung des Tubus nicht, muss ggf. auf invasive Nadelelektroden, die über das Op.-Feld in den gleichseitigen M. vocalis eingebracht werden, umgestiegen werden. In jedem Fall ist es essenziell, eine gute Ableitung der Mm. vocales über den N. vagus zu haben, bevor eventuelle iatrogene Schäden am N. laryngeus recurrens auftreten können.

Anschließend wird der obere Schilddrüsenpol freigelegt. Die Präparation erfolgt lateral überwiegend stumpf auf der Kapsel der Schilddrüse zwischen Kapsel und Grenzlamelle. Medial wird das Spatium cricopharyngeum eröffnet. Die oberen Polgefäße werden dargestellt. Meistens gelingt es, den Ramus externus des N. laryngeus superior, der den M. cricothyreoideus innerviert, mit dem Neuromonitoring zu identifizieren. Die oberen Polgefäße werden abgesetzt. Der Gefäßstamm wird kranial ligiert. Zuletzt haben wir gelegentlich auf eine Ligatur zugunsten eines versiegelnden Hilfsmittels (s. dort) verzichten können.

#### Laterale und kaudale Mobilisation der Schilddrüse

Im Weiteren wird von lateral nach medial auf der Schilddrüsenkapsel präpariert. Wichtig ist eine gute Exposition. Wir verwenden verschiedene Langenbeck-Haken und armierte Präpariertupfer. Verschiedene Gefäße müssen dabei versiegelt werden. Der untere Schilddrüsenpol wird zunächst angehoben. Dabei kommen die oberflächigen Anteile der sog. unteren Polgefäße zur Darstellung. Hier befindet sich i. d. R. die untere Nebenschilddrüse, die identifiziert und mit einer ausreichenden Blutzufuhr erhalten bleiben sollte. Zum Erhalt der Blutversorgung sollen die Kapselgefäße möglichst schilddrüsennah versiegelt werden. Ist der untere Pol ausreichend mobilisiert, lässt sich die Schilddrüse nach oben und medial in das Niveau des Hautschnitts mobilisieren. Auch bei sehr großen Strumen kann so die Exposition der hinteren Schilddrüsenanteile gelingen. Insbesondere sehr große Schilddrüsenknoten haben häufig eine gut erkennbare Kapsel, die stumpf (z. B. mit dem Finger) ausgelöst werden kann. Nur wenn die intrathorakalen Schilddrüsenanteile zu groß sind, um sie nach außen zu luxieren, kann zur Vermeidung einer Thorakotomie die Schilddrüsenkapsel kontrolliert eröffnet und die Schilddrüse intraglandulär verkleinert werden. Anschließend wird die Kapsel wieder vernäht.

#### Präparation der Schilddrüsenhinterseite

An dieser Stelle werden die untere Nebenschilddrüse und der N. laryngeus recurrens (NLR) aufgesucht. Letzteres ist gemäß Leitlinie gebotener Standard und gelingt mit dem Neuromonitoring zuverlässig. Zur Vermeidung von Traumata wird im Verlauf des NLR präpariert. Ist der Nerv identifiziert, wird er nach kranial bis zu seinem Abtauchen hinter den Ringknorpel von der Schilddrüse abpräpariert. Besondere Schwierigkeiten entstehen beim Vorliegen eines dorsalen Tuberkulums (Normvariante im Sinne einer Ausstülpung des Schilddrüsenlappens nach posterior, das sog. Tuberculum Zuckerkandl). Dieser Schilddrüsenanteil muss angehoben werden, um den Nerv verfolgen zu können. Vor dem Eintritt des Nervs hinter den Ringknorpel kann sich der NLR in maximal 4 Äste aufspalten. Hier, also am weitesten distal, ist der NLR am stärksten gefährdet. Sollte es bei der Präparation des NLR zu Blutungen kommen, ist der Nerv im Fall einer elektrischen Versieglung möglichst durch Auflage z. B. eines Präpariertupfers zu schonen, um ihn vor heißen Flüssigkeitströpfchen und anderer thermischer Schädigung zu schützen. Liegt der Nerv frei, kann die Schilddrüse abgesetzt werden. Anschließend ist die Funktion des NLR im Neuromonitoring zu prüfen und zu dokumentieren. Im Fall einer Schädigung gilt es zu erwägen, eine eventuell einzeitig geplante Operation der Gegenseite zu verschieben.

Das Schilddrüsenpräparat wird extrakorporal inspiziert, um eine eventuell versehentlich mitentfernte Nebenschilddrüse zu entdecken. Sind die Nebenschilddrüsen entfernt oder noch in situ, aber nicht mehr adäquat durchblutet, sollen sie – nach Verifizierung im Schnellschnitt – in Stückchen von 0,5–1 mm Kantenlänge in eine Tasche im M. sternocleidomastoideus reimplantiert werden (e73).

#### Wundverschluss und perioperative Betreuung

Es folgt eine subtile Blutstillung. Eine intrathorakale Druckerhöhung und eine medikamentöse Blutdruckerhöhung können helfen, eventuelle Blutungen zu identifizieren. Wird im Verlauf des N. laryngeus recurrens versiegelt, muss die Funktion noch einmal in Neuromonitoring überprüft werden. Bei Patienten mit erhöhtem Blutungsrisiko (z. B. Antikoagulanzien, Op. bei akuter Thyreoiditis o. Ä.) bringen wir Kollagenflies, Fibrinkleber oder Stärkepulver zur Blutstillung ein. Es folgt ggf. die Einlage einer 10er- oder 12er-Redon-Drainage pro Lobektomieseite. Der Wundverschluss erfolgt durch eine Adaptation der infrahyoidalen Muskulatur im Bereich der Linea alba, einer sorgfältigen Naht des Platysma und einer Subkutannaht. Die Haut wird heute geklebt. Es folgt ein Pflasterverband.

Eine Kontrolle der Schilddrüsenhormone hat aufgrund der langen Halbwertszeit i. d. R. Zeit, bis die definitive Histologie und damit die Entscheidung über eine eventuelle Radiojodtherapie vorliegt. Bei einer kompletten Thyreoidektomie müssen aber (auch bei makroskopisch sicherem Erhalt mindestens zweier Nebenschilddrüsen) noch am Op.-Tag Parathormon und Elektrolyte kontrolliert werden, da sonst innerhalb von Stunden eine Hypokalzämie droht, bei der ggf. Kalzium zu substituieren wäre.

#### Subtotale Lobektomien

Alle subtotalen Resektionen beginnen zunächst mit derselben Lagerung und denselben Schritten zur Freilegung der Schilddrüsenvorderseite. Danach kann auf Teilschritte je nach Umfang der geplanten Resektion verzichtet werden. Außer bei sehr weit anterioren und nahe der Trachea gelegenen Knoten wird aber eine Darstellung des N. laryngeus recurrens zu dessen sicherer Schonung empfohlen [28]. Im Zweifelsfall lässt sich die Funktion auch gut über eine Reizung des N. vagus prüfen, der insbesondere für den HNO-Arzt einfach aufzufinden ist. Für Teilresektionen werden versiegende Resektionsverfahren empfohlen, die Kapselnähte entbehrlich machen. Häufig kann bei intrakapsulären Knotenresektionen auf Drainagen verzichtet werden.

#### Alternative Zugangswege zur Schilddrüse

In den letzten Jahren haben sich zervikal minimal-invasive und extrazervikale Zugänge zur Schilddrüse entwickelt. Extrazervikale Zugangswege zur Schilddrüse haben alle das Ziel der Vermeidung sichtbarer Narben. Sie werden überwiegend in der asiatischen Welt eingesetzt.

##### Zervikale minimal-invasive Schilddrüsenchirurgie.

MIVAT steht für minimal-invasive, videoassistierte Thyreoidektomie. Erstbeschrieben wurde die MIVAT 1999 (e74) und hat sich weltweit verbreitet (e75, e76). Die Inzision ist deutlich kürzer (1,5–3 cm) als bei der konventionellen Chirurgie. Die ersten Op.-Schritte bis zur Darstellung der Schilddrüsenvorderseite sind der konventionellen Technik gleich. Dann startet das endoskopische Vorgehen. Empfohlen wird eine 30°-Optik mit einem Außendurchmesser von 5 mm. Für die Präparation und insbesondere die Gefäßversiegelungen werden versiegelnde Hilfsmittel verwendet. Op.-Schritte sind die Darstellung und Eröffnung der Linea alba, das Abschieben der geraden Halsmuskulatur von der Schilddrüse, Eingehen in die Schilddrüsenloge, Darstellen der Gefäß-Nerven-Scheide, erstes Neuromonitoring, Darstellen und Absetzen der oberen Polgefäße, Luxieren der Schilddrüse nach medial und ventral, Präparation des N. laryngeus recurrens (NLR) und der Nebenschilddrüsen, Überprüfung der Nervenfunktion mit dem Neuromonitoring, Durchtrennung des Ligamentum Berry und Komplettierung der Hemithyreoidektomie unter direkter Sicht (e77).

Das Vorgehen unterscheidet sich strukturell nicht von der konventionellen Thyreoidektomie. Es gelten die bekannten Prinzipien der Sichtdarstellung des NLR und der Autotransplantation der Nebenschilddrüsen. Als Vorteile werden bessere kosmetische Auswirkungen und geringere Schmerzen angeben. Die Komplikationsrate für Rekurrensparese, Nachblutungen und Hypokalzämien und die Kosten seien vergleichbar denen der konventionellen Chirurgie. Nachteilig ist eine längere Op.-Dauer (e75).

Durch die Schnittlänge ist die Größe einer zu resezierenden Schilddrüse auf 30 ml und der zu resezierenden Knoten auf 30 mm limitiert (e78). Im StuDoQ-Schilddrüsenregister (StuDoQ: Studien‑, Dokumentations- und Qualitätszentrum) wird der Anteil der MIVAT an den Schilddrüsenoperationen mit 2,2 % angegeben [18].

##### Extrazervikale endoskopische Thyreoidektomien.

Inzwischen wurde eine Vielzahl endoskopischer Zugangswege zur Schilddrüse beschrieben, etwa verschiedene Schnitte am anterioren oder lateralen Hals, der vorderen Brustwand oder retroaurikulär über einen sog. Face-Lift-Zugang (e79, e80). Nachstehend werden die beiden häufigsten dargestellt.

Beim „axillo-bilateral breast approach“ (ABBA) wird beidseits am Oberrand der Mamille inzidiert. Dieser Zugang ist wegen des subkutanen und Brustfettgewebes speziell für Frauen eignet. Von dort wird subkutan und subplatysmal bis auf Höhe des Schildknorpels präpariert. Anschließend werden die Trokare eingeführt. Es wird CO_2_ insuffliert, um Übersicht zu schaffen. Die Präparation erfolgt endoskopisch kontrolliert mit versiegelnden Instrumenten. Zusätzlich kann die ABBA-Technik beidseits um einen axillären Zugang erweitert werden. Eine gut bebilderte Technikbeschreibung findet sich bei Karakas (e77).

Indikationen sind benigne Schilddrüsenerkrankungen mit einem Volumen bis zu 40 ml, kleine differenzierte Karzinome bis 1 cm Größe oder follikuläre Läsionen bis zu 3 cm Größe (e81). Die berichteten Raten für postoperative Rekurrensparesen sollen der konventionellen Chirurgie vergleichbar sein. Allerdings muss über andere potenzielle Nervenschäden (N. axillaris, Armplexus) aufgeklärt werden (e82).

Beim axillären Zugang wird über eine Inzision in der vorderen Axillarlinie von lateral zum M. sternocleidomastoideus und durch diesen auf die Schilddrüse präpariert. In der Regel erfolgen 3 Inzisionen für 3 Trokare für das Endoskop und die Instrumente (e83). Bei einer beidseitigen Operation wird i. d. R. beiderseitig in der vorderen Axillarlinie inzidiert. Die chirurgischen Prinzipien sind dieselben. Die Op.-Zeiten sind gegenüber der konventionellen Operation etwa doppelt so lang (e84), die postoperativen Schmerzen vergleichbar (e85).

##### Roboterassistierte Thyreoidektomien.

Als Vorteile gelten die dreidimensionale Sicht, die 10-fache optische Vergrößerung und das tremorfreie Operieren. Auf die Grundzüge der robotorassistierten Chirurgie kann an dieser Stelle nicht eingegangen werden. Sie sind dem HNO-Arzt aus der Kopf-Hals-Tumor-Chirurgie bekannt. Es wird auf die Standardliteratur verwiesen. Roboterassistierte Thyreoidektomien werden entweder transaxillär (e86) oder neuerdings transoral (e87) oder retroaurikulär (e80) durchgeführt. Auch der ABBA-Zugang wurde schon mit Robotersystemen im Sinne eines „bilateral axillo-breast approach“ (BABA) kombiniert (e88). Nur die transorale Technik vermeidet jegliche Hautinzision.

Standard ist der transaxilläre Zugang mit weltweit über 10.000 Operationen (e75, e80). Bei der transaxillären roboterassistierten Thyreoidektomie (TRAT) wird über eine Inzision in der vorderen Axillarlinie von lateral zum M. sternocleidomastoideus und durch diesen auf die Schilddrüse präpariert. Durch Einsetzen eines geeigneten Sperrersystems wird der Op.-Situs exponiert, und es können bis zu 4 Roboterarme platziert werden. Grundsätzlich ist es möglich, nicht nur die ipsilaterale, sondern auch die kontralaterale Schilddrüse über diesen Zugang zu resezieren (e89, e90). In diesen Fällen wird empfohlen, eventuell einen Schilddrüsenrest im Bereich des Ligamentum Berry zu belassen, um den NLR nicht zu gefährden (e91).

Die Komplikationsraten werden als der konventionellen Chirurgie vergleichbar beschrieben. Kosten sind erheblich höher, Op.-Dauern sind länger. Als geeignet werden benigne Schilddrüsenknoten bis 3 cm Größe und Schilddrüsenlappen bis 40 ml Volumen beschrieben. Der Abstand zwischen Axilla und Jugulum soll maximal 17 cm betragen (e92).

Beim transoralen Vorgehen erfolgen 3 Inzisionen im Mundvorhof. Es werden lippenrotnahe Inzisionen zu Vermeidung von Nervenschäden am N. mentalis (V3) empfohlen. Nach der Platzierung der 3 Trokare erfolgen die einzelnen Op.-Schritte: Darstellung und Eröffnung der Linea alba, Durchtrennung des Schilddrüsenisthmus, Versorgung der oberen Polgefäße, Darstellung, Präparation und Neuromonitoring des NLR, Absetzen der Schilddrüse und Bergung über den mittleren oralen Zugang.

Geeignet sind Schilddrüsenlappen ≤ 6 cm oder ≤ 30 ml, benigne Knoten bis 6 cm, M. Basedow und – bedingt geeignet – kleinere differenzierte Schilddrüsenkarzinome. Als Ausschlusskriterien genannt werden fortgeschrittene, schlecht differenzierte und anaplastische Schilddrüsenmalignome, Lymphknotenmetastasen, Voroperationen am Hals, vorbestehende Rekurrensparese, retrosternale Lage der Schilddrüse und enorale Infektionen (e93).

#### Vorgehen bei malignen Tumoren

Das Prinzip der Strumektomie ist auch bei malignen Tumoren gleich. Je nach Entität und Tumorstadium ist zusätzlich eine Neck-Dissection erforderlich. Die Infiltration von Nachbargeweben kann weitere Op.-Schritte erforderlich machen. Diesbezüglich muss an dieser Stelle auf die Spezialliteratur verwiesen werden.

## Komplikationen und deren Management

Potenzielle Komplikationen nach einer Schilddrüsen-Op. können in Form von Nachblutungen, Wundinfektionen, Rekurrensparesen, Lymphfisteln und einem Hypoparathyreoidismus auftreten. Mit Ausnahme des Letzteren sind diese dem HNO-Chirurgen vertraut, weshalb an dieser Stelle auf die einschlägige HNO-Literatur verwiesen wird.

### Nachblutungen

Durch Einblutung ins Wundbett kann es zu einer potenziell bedrohlichen postoperativen Dyspnoe kommen. In der einschlägigen Literatur wird ein Wundset am Krankenbett empfohlen, welches die sofortige Wundöffnung zum Zweck der Drainage ermöglicht. Das Personal ist entsprechend zu unterweisen.

### Hypoparathyreoidismus

Die European Society of Endocrinology definiert den Hypoparathyreoidismus als Hypokalzämie mit inadäquat niedrigem Parathormon (e94). Im Fall einer permanenten Erkrankung kommt es wegen der kurzen Halbwertszeit des Parathormons zu teilweise erheblichen Einschränkungen der Lebensqualität. Das Leitsymptom besteht in einer Hypokalzämie mit Tetanie mit Spasmen im Bereich der Hände (Pfötchenstellung), der Gesichtsmuskulatur, des Larynx und der Atemmuskulatur teilweise mit subjektiver Dyspnoe. Frühsymptome sind Kribbelparästhesien an Fingern, Zehen und perioral sowie eine innere Unruhe der Patienten. Infolge einer vermehrten neuromuskulären Reizbarkeit kommt es beim Beklopfen der Wange zu Zuckungen am Mundwinkel (positives Chvostek-Zeichen) und zur Pfötchenstellung der Hand beim Aufpumpen der Blutdruckmanschette (Trousseau-Zeichen). Andere Symptome sind in Tab. 9 dargestellt.

**Tab. 9:** Klinische Manifestationen des permanenten Hypoparathyreoidismus. (Nach [29])

Organsystem	Befunde und Symptome
Renal	Nephrokalzinose, Nephrolithiasis, Niereninsuffizienz
Neuromuskulär	Missempfindungen perioral, Kribbelparästhesien, Tetanie, gesteigerter Sehnenreflex, Taubheit an den Extremitäten, Kraftlosigkeit, Myalgie
Ossär	Reduzierter Knochenumbau, Knochenschmerzen, vermehrte Knochendichte, Defekte am Zahnschmelz
Neurologisch	Übererregbarkeit, Depression, kognitive Einbußen („brain fog“), Müdigkeit, Verkalkungen, der Basalganglien (M. Fahr)
Kardiovaskulär	Arrhythmien, Palpitationen, Herzinsuffizienz
Okulär	Katarakt
Dermatologisch	Haarausfall, trockene Haut, Juckreiz, brüchige Nägel

Eindeutige Zahlen zur Häufigkeit des postoperativen Hypoparathyreoidismus gibt es offenbar nicht. Die Angaben variieren zwischen 1 und 50 % [29]. Je radikaler die Thyreoidektomie ausgeführt wird, desto mehr Nebenschilddrüsen sind in Gefahr, versehentlich mitreseziert zu werden, und desto häufiger tritt ein Hypoparathyreoidismus auf. Bei unilateralen Operationen kommt es in aller Regel nicht zu einem Hypoparathyreoidismus. Die Gefahr eines Hypoparathyreoidismus hängt außerdem von der Erfahrung des Chirurgen ab [30].

Aufgrund der kurzen Halbwertszeit des Parathormons von wenigen Minuten lässt sich der Funktionszustand postoperativ am besten durch Bestimmungen des Parathormons überprüfen. Die Hypokalzämie entwickelt sich oft erst während der ersten 48 h. Kommt es zu einem dauerhaften Hypoparathyreoidismus sind neben dem ionisierten Kalzium auch das albuminkorrigierte Kalzium, das Magnesium und das Phosphat zu überprüfen.

Die Therapie des Hypoparathyreoidismus soll einerseits die Symptomfreiheit und andererseits die Vermeidung von Folgeschäden bewirken. Insofern bedarf es immer der Beachtung der individuellen Symptomatik. Im Fall einer Tetanie werden 1–2 Ampullen 10%ige Kalziumlösung als Kurzinfusion über 10–20 min und anschließend weitere 6–8 Ampullen über 24 h verabreicht. Eine 2‑mal tägliche Kalziumbestimmung ist erforderlich, um die Dosierung anzupassen. Auf mögliche Herzrhythmusstörungen muss insbesondere bei Digitalismedikation geachtet werden. Ist der Patient wieder symptomfrei, wird die orale Substitution mit 1–3 g Kalzium per os in mehreren Dosen von maximal 500 mg eingestellt, da die maximale Kalziumaufnahme limitiert ist. Bei der Wahl des Präparats ist auf eine eventuelle Einnahme von Protonenpumpenhemmern zu achten, da Kalziumkarbonat das saure Magenmilieu für seine Resorption benötigt. Alternativen stehen mit Kalziumzitrat und Kalziumglukonat für diesen Fall zur Verfügung. Zur Verbesserung der Kalziumresorption soll frühzeitig 1,25-Dihydroxy-Cholecalcitriol in einer Dossierung von 2‑mal 0,25–1,0 µg eingesetzt werden. Ein möglicher Magnesiummangel sollte ebenfalls substituiert werden, da Magnesium die Parathormonproduktion und dessen Wirkung am Zielgewebe beeinflusst. Im Fall einer Hyperkalzurie sollen Thiaziddiuretika zur Anwendung kommen.

Bei Patienten mit einem permanenten Hypoparathyreoidismus, die mit der beschrieben Substitutionstherapie nicht gut einzustellen sind, kann heute rekombinantes humanes Parathormon eingesetzt werden (e95). Die Applikation erfolgt subkutan und bedarf eines intensiven Monitorings von Klinik und Laborparametern. Die Tab. 10 fasst die Therapieziele bei permanentem Hypoparathyreoidismus (e94) zusammen.

**Tab. 10:** Therapieziele in der Behandlung des permanenten Hypoparathyreoidismus (e94; hier nach [29])

1) Albuminkorrigierter Kalziumwert im unteren Normbereich oder knapp unterhalb des Referenzbereichs bei gleichzeitiger Symptomfreiheit des Patienten
2) Kalziumausscheidung im 24-h-Sammelurin im geschlechtsspezifischen Normbereich
3) Serumphosphatspiegel im Referenzbereich
4) Kalzium-Phosphat-Produkt < 4,4 mmol^2^/l^2^ bzw. < 55 mg^2^/dl^2^
5) Magnesiumspiegel im Referenzbereich
6) Adäquater Vitamin-D-Spiegel
7) Personalisierte Behandlung erforderlich mit Fokus auf Lebensqualität und Wohlbefinden
8) Ausreichende Information und Schulung des Patienten erforderlich

## Fazit für die Praxis

- Die Schilddrüse ist ein komplexes Organ, und seine Behandlung geht weit über eine einfache Strumektomie hinaus.
- Gerade vor dem Spannungsfeld Standardisierung vs. Individualisierung sind für den HNO-Arzt gute Kenntnisse und Kompetenzen zum Thema Schilddrüse wichtig.
- Nur so können wir im Sinne unserer Patienten sinnvoll mit den Ressourcen im Gesundheitswesen umgehen.
- Die Autoren empfehlen und leben eine gute interdisziplinäre Vernetzung mit allen an der Schilddrüse beteiligten Fachgebieten und wünschen dem Leser viel Erfolg in diesem uns beiden sehr am Herzen liegenden Teilgebiet.

## Supplementary Information

Weiterführende Literatur „(e1)“ usw. = Verweis auf weiterf. Lit. im ESM

## References

[1] Ostrowski P, Bonczar M, Iwanaga J, Michalczak M, Dziedzic M, Del Carmen Yika A, Gil A, Sporek M, Szczepanek E, Niemczyk K, Walocha J, Koziej M (2023) The prevalence and anatomy of the pyramidal lobe of the thyroid gland: A meta-analysis with implications for thyroid surgery. Clin Anat 36(6):937–945 (Sep) 37245093 10.1002/ca.24062

[2] Goichot B, Caron P, Landron F, Bouée S (2016) Clinical presentation of hyperthyroidism in a large representative sample of outpatients in France: relationships with age, aetiology and hormonal parameters. Clin Endocrinol (oxf) 84(3):445–451 (Mar) 25959282 10.1111/cen.12816

[3] de Leo S, Lee SY, Braverman LE (2016) Hyperthyroidism. Lancet 388(10047):906–918 (27;) 27038492 10.1016/S0140-6736(16)00278-6 PMC5014602

[4] Maurer E, Hyperthyreose HK (2023) In: Bartsch DK, Holzer K (Hrsg) Endokrine Chirurgie. Springer, Berlin, S 99–116

[5] Garmendia Madariaga A, Palacios SS, Guillén-Grima F, Galofré JC (2014) The incidence and prevalence of thyroid dysfunction in Europe: a meta-analysis. J Clin Endocrinol Metab 99(3):923–931 (Mar) 24423323 10.1210/jc.2013-2409

[6] Brent GA (2008) Clinical practice. Graves’ disease. N Engl J Med 358(24):2594–2605 (Jun 12) 18550875 10.1056/NEJMcp0801880

[7] Galindo RJ, Hurtado CR, Pasquel FJ, García TR, Peng L, Umpierrez GU (2019) National Trends in Incidence, Mortality, and Clinical Outcomes of Patients Hospitalized for Thyrotoxicosis With and Without Thyroid Storm in the United States, 2004–2013. Thyroid 29(1):36–43 (Jan) 30382003 10.1089/thy.2018.0275 PMC6916241

[8] Synoracki S, Ting S, Schmid KW (2016) Entzündungen der Schilddrüse. Pathologe 37(3):215–223 (May) 27100868 10.1007/s00292-016-0157-9

[9] Dong YH, Fu DG (2014) Autoimmune thyroid disease: mechanism, genetics and current knowledge. Eur Rev Med Pharmacol Sci 18(23):3611–3618 25535130

[10] Hollowell JG, Staehling NW, Flanders WD, Hannon WH, Gunter EW, Spencer CA, Braverman LE (2002) Serum TSH, T(4), and thyroid antibodies in the United States population (1988 to 1994): National Health and Nutrition Examination Survey (NHANES III). J Clin Endocrinol Metab 87(2):489–499 (Feb) 11836274 10.1210/jcem.87.2.8182

[11] Caturegli P, de Remigis A, Rose NR (2014) Hashimoto thyroiditis: clinical and diagnostic criteria. Autoimmun Rev 13(4-5):391–397 24434360 10.1016/j.autrev.2014.01.007

[12] Musholt TJ, Clerici T, Dralle H, Frilling A, Goretzki PE, Hermann MM, Kussmann J, Lorenz K, Nies C, Schabram J, Schabram P, Scheuba C, Simon D, Steinmüller T, Trupka AW, Wahl RA, Zielke A, Bockisch A, Karges W, Luster M, Schmid KW (2011) Interdisciplinary Task Force Guidelines of the German Association of Endocrine Surgeons. German Association of Endocrine Surgeons practice guidelines for the surgical treatment of benign thyroid disease. Langenbecks Arch Surg 396(5):639–649 (Jun) 21424798 10.1007/s00423-011-0774-y

[13] Schmidt KW (2023) Pathologie der benignen und malignen Schilddrüsenveränderungen – was der endokrine Chirurg wissen sollte. In: Bartsch DK, Holzer K (Hrsg) Endokrine Chirurgie. Springer, Berlin, S 11–32

[14] Japan Thyroid Association Guidelines for the diagnosis of chronic thyreoiditis. https://www.japanthyroid.jp/en/guidelines.html (Hashimoto disease)

[15] Tuttle RM, Haugen B, Perrier ND (2017) Updated American Joint Committee on Cancer/Tumor-Node-Metastasis Staging System for Differentiated and Anaplastic Thyroid Cancer (Eighth Edition): What Changed and Why? Thyroid 27(6):751–756 28463585 10.1089/thy.2017.0102 PMC5467103

[16] International Agency for Research on Cancer (Hrsg.) (2020) World Cancer report: Cancer research for cancer preservation. In: Lyon. ISBN 978-92-832-0447‑3

[17] Dralle H (2019) Die neue TNM-Klassifikation der Schilddrüsenkarzinome. Chirurg 90(Suppl 2):101 (Mar) 30758554 10.1007/s00104-019-0881-9

[18] Bartsch DK, Dotzenrath C, Vorländer C, Zielke A, Weber T, Buhr HJ, Klinger C, Lorenz K (2019) The StuDoQ/Thyroid Study The StuDoQ/Thyroid Study Group. Current Practice of Surgery for Benign Goitre—An Analysis of the Prospective DGAV StuDoQ Thyroid Registry. J Clin Med 8(4):477 30965665 10.3390/jcm8040477 PMC6517925

[19] Dotzenrath C, Holzer K, Lorenz K, Musholt TJ, Vorländer C, Chirurgische Arbeitsgemeinschaft Endokrinologie (CAEK) (federführend) der Deutschen Gesellschaft für Allgemein- und Viszeralchirurgie (DGAV) (2021) S2k-Leitlinie Operative Therapie benigner Schilddrüsenerkrankungen

[20] Durante C, Hegedüs L, Czarniecka A, Paschke R, Russ G, Schmitt F, Soares P, Solymosi T, Papini E (2023) 2023 European Thyroid Association Clinical Practice Guidelines for thyroid nodule management. Eur Thyroid J 12(5):e230067 37358008 10.1530/ETJ-23-0067 PMC10448590

[21] Morris LF, Ragavendra N, Yeh MW (2008) Evidence-based assessment of the role of ultrasonography in the management of benign thyroid nodules. World J Surg 32:1253–1263 18311500 10.1007/s00268-008-9494-z

[22] Haugen BR, Alexander EK, Bible KC, Doherty GM, Mandel SJ, Nikiforov YE, Pacini F, Randolph GW, Sawka AM, Schlumberger M, Schuff KG, Sherman SI, Sosa JA, Steward DL, Tuttle RM, Wartofsky L (2016) 2015 American Thyroid Association Management Guidelines for Adult Patients with Thyroid Nodules and Differentiated Thyroid Cancer: The American Thyroid Association Guidelines Task Force on Thyroid Nodules and Differentiated Thyroid Cancer. Thyroid 26(1):1–133 26462967 10.1089/thy.2015.0020 PMC4739132

[23] Russ G, Bonnema SJ, Erdogan MF, Durante C, Ngu R, Leenhardt L (2017) European Thyroid Association Guidelines for Ultrasound Malignancy Risk Stratification of Thyroid Nodules in Adults: The EU-TIRADS. Eur Thyroid J 6(5):225–237 (Sep) 29167761 10.1159/000478927 PMC5652895

[24] Filetti S, Durante C, Hartl D, Leboulleux S, Locati LD, Newbold K, Papotti MG, Berruti A, Guidelines Committee ESMO (2019) Thyroid cancer: ESMO Clinical Practice Guidelines for diagnosis, treatment and follow-up. Ann Oncol. In: 30(12). Dec, Bd. 1, S 1856–1883 10.1093/annonc/mdz400 31549998

[25] Périé S, Santini J, Kim HY, Dralle H, Randolph GW (2018) International consensus (ICON) on comprehensive management of the laryngeal nerves risks during thyroid surgery. Eur Ann Otorhinolaryngol Head Neck Dis 135(1S):S7–S10 (Feb) 29361440 10.1016/j.anorl.2017.11.010

[26] Randolph GW, Dralle H; International Intraoperative Monitoring Study Group, Abdullah H, Barczynski M, Bellantone R, Brauckhoff M, Carnaille B, Cherenko S, Chiang FY, Dionigi G, Finck C, Hartl D, Kamani D, Lorenz K, Miccolli P, Mihai R, Miyauchi A, Orloff L, Perrier N, Poveda MD, Romanchishen A, Serpell J, Sitges-Serra A, Sloan T, van Slycke S, Snyder S, Takami H, Volpi E, Woodson G (2011) Electrophysiologic recurrent laryngeal nerve monitoring during thyroid and parathyroid surgery: international standards guideline statement. Laryngoscope 121(1):1–16 10.1002/lary.21119 21181860

[27] Ruggiero R, Docimo G, Bosco A, Lanza Volpe M, Terracciano G, Gubitosi A, Docimo L (2018) Update on sutureless thyroidectomy. G Chir 39(1):45–50 29549681 10.11138/gchir/2018.39.1.045 PMC5902144

[28] Zielke A, Operationstechnik SCA (2023) konventionelle Schilddrüsenresektion bei benigner Struma. In: Bartsch DK, Holzer K (Hrsg) Endokrine Chirurgie. Springer, Berlin, S 41–47

[29] Schabram J (2023) Management postoperativer Komplikationen der Schilddrüsenchirurgie. In: Bartsch DK, Holzer K (Hrsg) Endokrine Chirurgie. Springer, Berlin, S 227–239

[30] Dralle H, Lorenz K, Machens A (2011) State of the art: surgery for endemic goiter—a plea for individualizing the extent of resection instead of heading for routine total thyroidectomy. Langenbecks Arch Surg 396(8):1137–1143 (Dec) 21630080 10.1007/s00423-011-0809-4

