# Supplementary material for: Diagnosis and surgical therapy of thyroid diseases
Source: HNO. 2025 Apr 9;73(6):401–22. [Article in German] doi: 10.1007/s00106-025-01620-5 (PMC12101998; doi:10.1007/s00106-025-01620-5)
Supplement: Supplementary file 1 — Weiterführende Literatur „(e1)“ usw. = Verweis auf weiterf. Lit. im ESM [file 106_2025_1620_MOESM1_ESM.pdf]

**Supplementary Information**

**Weiterführende Literatur:**


e1. Burger F, Fritsch H, Zwierzina ME, Prommegger R, Konschake M. Postoperative Hypoparathyroidism in Thyroid Surgery: Anatomic-Surgical Mapping of the Parathyroids and Implications for Thyroid Surgery. Sci Rep. 2019 Oct 30;9(1):15700

e2. Prommegger R, Konschake M. Chirurgische Anatomie der Schilddrpse. In Bartsch DK, Holzer K (Hrsg.). Endokrine Chirurgie. Springer Verlag Berlin, 2023, S 33-39

e3. Konschake M, Zwierzina ME, Pechriggl EJ, Moriggl B, Brenner E, Hörmann R, Prommegger R. The nonrecurrent laryngeal nerve: A clinical anatomic mapping with regard to intraoperative neuromonitoring. Surgery. 2016 Jul;160(1):161-168

e4. Gemsenjäger E. Das Bild der Grenzlamelle in der Schilddrüsenchirurgie. Chirurg. 2009 Dec;80(12):1165

e5. Konschake M, Burger F, Zwierzina ME. Peripheral Nerve Anatomy Revisited: Modern Requirements for Neuroimaging and Microsurgery. Anat Rec (Hoboken). 2019 Aug;302(8):1325-1332

e6. Mohebati A, Shaha AR. Anatomy of thyroid and parathyroid glands and neurovascular relations. Clin Anat. 2012 Jan;25(1):19-31

e7. Herold G. Innere Medizin 2020. Walter de Gryter Verlag Berlin. ISBN-10 3981466098

e8. Deutsches Schilddrüsenzentrum. https://www.deutsches-schilddruesenzentrum.de/

e9. Zieren HU. Diagnostik und Therapie des Morbus Basedow. (https://www.deutsches-schilddruesenzentrum.de)

e10. Selmer C, Olesen JB, Hansen ML, von Kappelgaard LM, Madsen JC, Hansen PR, Pedersen OD, Faber J, Torp-Pedersen C, Gislason GH. Subclinical and overt thyroid dysfunction and risk of all-cause mortality and cardiovascular events: a large population study. J Clin Endocrinol Metab. 2014 Jul;99(7):2372-82

e11. Abe E, Marians RC, Yu W, Wu XB, Ando T, Li Y, Iqbal J, Eldeiry L, Rajendren G, Blair HC, Davies TF, Zaidi M. TSH is a negative regulator of skeletal remodeling. Cell. 2003 Oct 17;115(2):151-62

e12. Krassas GE, Poppe K, Glinoer D. Thyroid function and human reproductive health. Endocr Rev. 2010 Oct;31(5):702-55

e13. Vejbjerg P, Knudsen N, Perrild H, Laurberg P, Carlé A, Bülow Pedersen I, Rasmussen LB, Ovesen L, Jørgensen T. Lower prevalence of mild hyperthyroidism related to a higher iodine intake in the population: prospective study of a mandatory iodization programme. Clin Endocrinol (Oxf). 2009 Sep;71(3):440-5

e14. Nyström HF, Jansson S, Berg G. Incidence rate and clinical features of hyperthyroidism in a long-term iodine sufficient area of Sweden (Gothenburg) 2003-2005. Clin Endocrinol (Oxf) . 2013 May;78(5):768-76

e15. Utiger RD. Cigarette smoking and the thyroid. N Engl J Med. 1995 Oct 12;333(15):1001-2.

e16. Wiersinga WM. Smoking and thyroid. Clin Endocrinol (Oxf) . 2013 Aug;79(2):145-51

e17. Marinò M, Latrofa F, Menconi F, Chiovato L, Vitti P. Role of genetic and non-genetic factors in the etiology of Graves' disease. J Endocrinol Invest. 2015 Mar;38(3):283-94

e18. Bartalena L. Diagnosis and management of Graves disease: a global overview. Nat Rev Endocrinol. 2013 Dec;9(12):724-34

e19. Prasek K, Płazińska MT, Królicki L. Diagnosis and treatment of Graves' disease with particular emphasis on appropriate techniques in nuclear medicine. General state of knowledge. Nucl Med Rev Cent East Eur. 2015;18(2):110-6
e20. Laurberg P, Berman DC, Bülow Pedersen I, Andersen S, Carlé A. Incidence and clinical presentation of moderate to severe graves' orbitopathy in a Danish population before and after iodine fortification of salt. J Clin Endocrinol Metab. 2012 Jul;97(7):2325-32
e21. Bartalena L, Kahaly GJ, Baldeschi L, Dayan CM, Eckstein A, Marcocci C, Marinò M, Vaidya B, Wiersinga WM; EUGOGO †. The 2021 European Group on Graves' orbitopathy (EUGOGO) clinical practice guidelines for the medical management of Graves' orbitopathy. Eur J Endocrinol. 2021 Aug 27; 185(4): G43-G67
e22. Akamizu T, Satoh T, Isozaki O, Suzuki A, Wakino S, Iburi T, Tsuboi K, Monden T, Kouki T, Otani H, Teramukai S, Uehara R, Nakamura Y, Nagai M, Mori M; Japan Thyroid Association. Diagnostic criteria, clinical features, and incidence of thyroid storm based on nationwide surveys. Thyroid. 2012 Jul;22(7):661-79
e23. van Soestbergen MJ, van der Vijver JC, Graafland AD. Recurrence of hyperthyroidism in multinodular goiter after long-term drug therapy: a comparison with Graves' disease. J Endocrinol Invest. 1992 Dec;15(11):797-800
e24. Moleti M, Violi MA, Montanini D, Trombetta C, di Bella B, Sturniolo G, Prestin S, Alibrandi A, Campennì A, Baldari S, Trimarchi F, Vermiglio F. Radioiodine ablation of postsurgical thyroid remnants after treatment with recombinant human TSH (rhTSH) in patients with moderate-to-severe graves' orbitopathy (GO): a prospective, randomized, single-blind clinical trial. J Clin Endocrinol Metab. 2014 May;99(5):1783-9
e25. Dietlein M, Grünwald F, Schmidt M, Kreissl MC, Luster M; Deutsche Gesellschaft für Allgemein- und Viszeralchirurgie e. V. (DGAV); Chirurgische Arbeitsgemeinschaft Endokrinologie (CAEK) der DGAV; Deutsche Gesellschaft für Chirurgie e. V. (DGCH); Deutsche Gesellschaft für Endokrinologie e. V. (DGE). DGN-Handlungsempfehlung (S1-Leitlinie) Radioiodtherapie bei benignen Schilddrüsenerkrankungen. Stand 6/2022 – AWMF Nr. 031-003. Nuklearmedizin. 2024 Feb;63(1):8-20
e26. Haugen BR, Alexander EK, Bible KC, Doherty GM, Mandel SJ, Nikiforov YE, Pacini F, Randolph GW, Sawka AM, Schlumberger M, Schuff KG, Sherman SI, Sosa JA, Steward DL, Tuttle RM, Wartofsky L. 2015 American Thyroid Association Management Guidelines for Adult Patients with Thyroid Nodules and Differentiated Thyroid Cancer: The American Thyroid Association Guidelines Task Force on Thyroid Nodules and Differentiated Thyroid Cancer. Thyroid. 2016 Jan;26(1):1-133
e27. Pearce EN, Farwell AP, Braverman LE. Thyroiditis. N Engl J Med. 2003 Jun 26;348(26):2646-55
e28. Pyzik A, Grywalska E, Matyjaszek-Matuszek B, Roliński J. Immune disorders in Hashimoto's thyroiditis: what do we know so far? J Immunol Res. 2015:2015:979167
e29. Desailloud R, Hober D. Viruses and thyroiditis: an update. Virol J. 2009 Jan 12:6:5
e30. Barbesino G, Chiovato L. The genetics of Hashimoto's disease. Endocrinol Metab Clin North Am. 2000 Jun;29(2):357-74
e31. Heiberg Brix T, Hegedüs L, Gardas A, Banga JP, Nielsen CH. Monozygotic twin pairs discordant for Hashimoto's thyroiditis share a high proportion of thyroid peroxidase autoantibodies to the immunodominant region A. Further evidence for genetic transmission of epitopic "fingerprints". Autoimmunity. 2011 May;44(3):188-94
e32. Ajjan RA, Weetman AP. The Pathogenesis of Hashimoto's Thyroiditis: Further Developments in our Understanding. Horm Metab Res. 2015 Sep;47(10):702-10

e33. Weetman AP. An update on the pathogenesis of Hashimoto's thyroiditis. J Endocrinol Invest. 2021 May;44(5):883-890
e34. Jin B, Wang S, Fan Z. Pathogenesis Markers of Hashimoto's Disease-A Mini Review. Front Biosci (Landmark Ed) . 2022 Oct 31;27(10):297
e35. Lee SY, Pearce EN. Assessment and treatment of thyroid disorders in pregnancy and the postpartum period. Nat Rev Endocrinol. 2022 Mar;18(3):158-171
e36. Gao X, Wang X, Han Y, Wang H, Li J, Hou Y, Yang Y, Wang H, Teng W, Shan Z. Postpartum Thyroid Dysfunction in Women With Known and Newly Diagnosed Hypothyroidism in Early Pregnancy. Front Endocrinol (Lausanne) . 2021 Nov 26:12:746329
e37. Pearce EN. Management of thyreotxicosis: preconception, pregnancy, and the postpartum period. Endocr Pract. 2019 Jan;25(1):62-68
e38. Tutal E, Ozaras R, Leblebicioglu H. Systematic review of COVID-19 and autoimmune thyroiditis. Travel Med Infect Dis. 2022 May-Jun:47:102314
e39. Kravets I. Hyperthyroidism: Diagnosis and Treatment. Am Fam Physician. 2016 Mar 1;93(5):363-70
e40. Majety P, Hennessey JV. Acute and Subacute, and Riedel's Thyroiditis. In: Feingold KR, Anawalt B, Blackman MR, Boyce A, Chrousos G, Corpas E, de Herder WW, Dhatariya K, Dungan K, Hofland J, Kalra S, Kaltsas G, Kapoor N, Koch C, Kopp P, Korbonits M, Kovacs CS, Kuohung W, Laferrère B, Levy M, McGee EA, McLachlan R, New M, Purnell J, Sahay R, Shah AS, Singer F, Sperling MA, Stratakis CA, Trence DL, Wilson DP: Endotext [Internet]. South Dartmouth (MA): MDText.com, Inc.; 2000. Updated 2022
e41. Pan XF, Gu JQ, Shan ZY. Increased risk of thyroid autoimmunity in rheumatoid arthritis: a systematic review and meta-analysis. Endocrine. 2015 Sep;50(1):79-86
e42. Mizukoshi T, Noguchi S, Murakami T, Futata T, Yamashita H. Evaluation of recurrence in 36 subacute thyroiditis patients managed with prednisolone. Intern Med. 2001 Apr;40(4):292-5Fu-shun Pan, Wei Wang, Yan Wang, Ming Xu, Jin-yu Liang, Yan-ling Zheng, Xiao-yan Xie, Xiao-xi Li. Sonographic features of thyroid nodules that may help distinguish clinically atypical subacute thyroiditis from thyroid malignancy. J Ultrasound Med. 2015 Apr;34(4):689-96
e43. Bahadir CT, Yilmaz M, Kiliçkan E. Factors affecting recurrence in subacute granulomatous thyroiditis. Arch Endocrinol Metab. 2022 May 12;66(3):286-294
e44. Lüers JC, Hoffmann B, Koerber F, Rahimi G, Streppel M. Sinus-piriformis-Fistel als seltene Ursache von dyspnoe bei einem Neugeborenen. HNO. 2008 Sep;56(9):975-80
e45. Theurer S, Siebolts U, Lorenz K, Dralle H, Schmid KW. Ektopes Gewebe der Schilddrüse und der Nebenschilddrüsen. Pathologe 2018 Sep;39(5):379-389
e46. Gopal RA, Acharya SV, Bandgar T, Menon PS, Marfatia H, Shah NS. Clinical profile of ectopic thyroid in Asian Indians: a single-center experience. Endocr Pract. 2009 May-Jun;15(4):322-5
e47. Iftikhar H, Ikram M, Rizwan Nathani K, Yar Muhammad A. Papillary. Thyroid Carcinoma within Thyroglossal Duct Cyst: Case Series and Literature Review. Int Arch Otorhinolaryngol. 2018 Jul;22(3):253-255
e48. Peres C, Rombo N, Guia Lopes L, Simões C, Roque R. Thyroglossal Duct Cyst Carcinoma With Synchronous Thyroid Papillary Carcinoma: A Case Report and Literature Review. Cureus. 2022 Aug 30;14(8):e28570
e49. de Oliveira Filho JR, de Nadai TR. Parasitic thyroid nodules in patient with nontoxic multinodular goiter: a case report. J Med Case Rep. 2014 Feb 20:8:66
e50. Baker LJ, Gill AJ, Chan C, Lin BP, Crawford BA. Parasitic thyroid nodules: cancer or not? Endocrinol Diabetes Metab Case Rep. 2014:2014:140027

e51. Kairanna N, Vasudevan G, Karanth V, Sharan K. Ectopic Cervical Thymoma: An Uncommon Entity. Indian J Otolaryngol Head Neck Surg. 2022 Dec;74(Suppl 3):5884-5887
e52. Williams ED, Toyn CE, Harach HR. The ultimobranchial gland and congenital thyroid abnormalities in man. J Pathol. 1989 Oct;159(2):135-41
e53. Husson O, Haak HR, van Steenbergen LN, Nieuwlaat WA, van Dijk BA, Nieuwenhuijzen GA, Karim-Kos H, Kuijpens JL, van de Poll-Franse LV, Coebergh JW. Rising incidence, no change in survival and decreasing mortality from thyroid cancer in The Netherlands since 1989. Endocr Relat Cancer. 2013 Mar 26;20(2):263-71
e54. Robert Koch-Institut, Gesellschaft der epidemiologischen Krebsregister in Deutschland. Krebs in Deutschland 2015/2016. 12. Ausgabe 2019. doi: 10.25646/5977
e55. Kitahara CM, Sosa JA. The changing incidence of thyroid cancer. Nat Rev Endocrinol. 2016 Nov;12(11):646-653
e56. Hildesheim S. Entitäten des Schilddrüsenmalignoms. Auswertung der deutschen und europäischen Datenbanken. Diplomarbeit. Semmelweis Universität, Medizinische Fakultät, Asklepios Campus Hamburg. 2021.
e57. Kleinhans H, Kaptur S, Verse T. Chirurgische Therapie einer persistierenden Diarrhoe. HNO. 2012 Apr;60(4):348-51
e58. Richter D, Müller S, Mantsopoulos K, Koch M, Iro H, Sievert M. Sonographische Beurteilung des Schilddrüsenknotens – Wunsch oder Realität? Forum HNO 2024; 26: 68-79
e59. Dralle H, Sekulla C, Lorenz K, Brauckhoff M, Machens A; German IONM Study Group. Intraoperative monitoring of the recurrent laryngeal nerve in thyroid surgery. World J Surg. 2008 Jul;32(7):1358-66
e60. Schneider R, Machens A, Sekulla C, Lorenz K, Elwerr M, Dralle H. Superiority of continuous over intermittent intraoperative nerve monitoring in preventing vocal cord palsy. Br J Surg. 2021 May 27;108(5):566-573
e61. Schneider R, Machens A, Randolph GW, Kamani D, Lorenz K, Dralle H. Opportunities and challenges of intermittent and continuous intraoperative neural monitoring in thyroid surgery. Gland Surg. 2017 Oct;6(5):537-545
e62. Schneider R, Machens A, Lorenz K, Dralle H. Intraoperative nerve monitoring in thyroid surgery-shifting current paradigms. Gland Surg. 2020 Feb;9(Suppl 2):S120-S128
e63. Dralle H, Lorenz K, Schabram P, Musholt TJ, Dotzenrath C, Goretzki PE, Kußmann J, Niederle B, Nies C, Schabram J, Scheuba C, Simon D, Steinmüller T, Trupka A. Intraoperatives Neuromonitoring in der Schilddrüsenchirurgie. Empfehlungen der Chirurgischen Arbeitsgemeinschaft Endokrinologie. Chirurg. 2013 Dec;84(12):1049-56
e64. Lorenz K, Abuazab M, Sekulla C, Schneider R, Nguyen Thanh P, Dralle H. Results of intraoperative neuromonitoring in thyroid surgery and preoperative vocal cord paralysis. World J Surg. 2014 Mar;38(3):582-91
e65. Coerper S, Stengl W. Einführung der Gefäßversiegelung in einem Schilddrüsenzentrum: Risiken und wirtschaftlicher Nutzen. Zenttralbl Chir 2018; 143(5): 543-9
e66. Zanghì A, Cavallaro A, di Vita M, Cardì F, di Mattia P, Piccolo G, Barbera G, Urso M, Cappellani A. The safety of the Harmonic® FOCUS in open thyroidectomy: a prospective, randomized study comparing the Harmonic® FOCUS and traditional suture ligation (knot and tie) technique. Int J Surg. 2014:12 Suppl 1:S132-5
e67. Teksoz S, Bukey Y, Ozcan M, Arikan AE, Ozyegin A. Sutureless thyroidectomy with energy-based devices: Cerrahpasa experience. Updates Surg. 2013 Dec;65(4):301-7

e68. Arora RD, Prajwal SD, Nagaraja Rao K, Singh A, Nagarkar NM, Abishek AP. A Comparison of Harmonic Scalpel and Conventional Techniques for Thyroidectomy. Indian J Otolaryngol Head Neck Surg. 2023 Dec;75(4):3410-3414
e69. O'Neill CJ, Chang LY, Suliburk JW, Sidhu SB, Delbridge LW, Sywak MS. Sutureless thyroidectomy: surgical technique. ANZ J Surg. 2011 Jul-Aug;81(7-8):515-8
e70. Schütte L. Einsatz des Ultraschallskalpells in der Schilddrüsenchirurgie – sinnvolle Entwicklung? Diplomarbeit Semmelweis Universität, Medizinische Fakultät, Asklepios Campus Hamburg. 2021
e71. Spartalis E, Giannakodimos A, Giannakodimos I, Ziogou A, Papasilekas T, Patelis N, Schizas D, Troupis T. The role of LigaSure™ and Harmonic Scalpel in the preservation of recurrent laryngeal nerve during thyroidectomy. Ann R Coll Surg Engl. 2022 May;104(5):324-329
e72. van Beurden F, van Willigen DM, Vojnovic B, van Oosterom MN, Brouwer OR, van der Poel HG, Kobayashi H, van Leeuwen WB jr., Buckle T. Multi-Wavelength Fluorescence in Image-Guided Surgery, Clinical Feasibility and Future Perspectives. Mol Imaging. 2020 Jan-Dec:19:1536012120962333
e73. Hicks G, George R, Sywak M. Short and long-term impact of parathyroid autotransplantation on parathyroid function after total thyroidectomy. Gland Surg. 2017 Dec;6(Suppl 1):S75-S85
e74. Miccoli P, Berti P, Conte M, Bendinelli C, Marcocci C. Minimally invasive surgery for thyroid small nodules: preliminary report. J Endocrinol Invest. 1999 Dec;22(11):849-51
e75. de Vries LH, Aykan D, Lodewijk L, Damen JA, Borel Rinkes IH, Vriens MR. Outcomes of Minimally Invasive Thyroid Surgery - A Systematic Review and Meta-Analysis. Front Endocrinol (Lausanne). 2021 Aug 12:12:719397
e76. Miccoli P, Fregoli L, Rossi L, Papini P, Ambrosini CE, Bakkar S, De Napoli L, Aghababyan A, Matteucci V, Materazzi G. Minimally invasive video-assisted thyroidectomy (MIVAT). Gland Surg. 2020 Jan;9(Suppl 1):S1-S5
e77. Karakas E, Maurer E. Operationstechnik: alternative Zugangswege zur Schilddrüse (MIVAT, ABBA, TRAT, TOETVA). In Bartsch DK, Holzer K (Hrsg.). Endokrine Chirurgie. Springer Verlag Berlin, 2023, S 49-63
e78. Pisanu A, Podda M, Reccia I, Porceddu G, Uccheddu A. Systematic review with meta-analysis of prospective randomized trials comparing minimally invasive video-assisted thyroidectomy (MIVAT) and conventional thyroidectomy (CT). Langenbecks Arch Surg. 2013 Dec;398(8):1057-68
e79. Slotema ET, Sebag F, Henry JF. What is evidence for endoscopic thyroidectomy in the management of bening thyroid disease? World J Surg. 2008;32:1325-32
e80. Trahan J, Pelaez L, DiLeo M, Nuss DW, Son LS, Walvekar RR. Retro-Auricular Thyroidectomy: An Open Approach. Indian J Otolaryngol Head Neck Surg. 2018 Jun;70(2):218-222
e81. Bärlehner E, Benhidjeb T. Cervical scarless endoscopic thyroidectomy: Axillo-bilateral-breast approach (ABBA). Surg Endosc. 2008 Jan;22(1):154-7
e82. Choi JY, Lee KE, Chung KW, Kim SW, Choe JH, Koo DH, Kim Sj, Lee J, Chung YS, Oh SK, Youn YK. Endoscopic thyroidectomy via bilateral axillo-breast approach (BABA): review of 512 cases in a single institute. Surg Endosc. 2012 Apr;26(4):948-55
e83. Ikeda Y, Takami H, Sasaki Y, Kan S, Niimi M. Endoscopic neck surgery by the axillary approach. J Am Coll Surg. 2000 Sep;191(3):336-40
e84. Ikeda Y, Takami H, Sasaki Y, Takayama J, Niimi M, Kan S. Comparative study of thyroidectomies. Endoscopic surgery versus conventional open surgery Surg Endosc. 2002 Dec;16(12):1741-5
e85. Ikeda Y, Takami H, Sasaki Y, Takayama J, Niimi M, Kan S. Clinical benefits in endoscopic thyroidectomy by the axillary approach. J Am Coll Surg. 2003 Feb;196(2):189-95

e86. Kang SW, Lee SC, Lee SH, Lee KY, Jeong JJ, Lee YS, Nam KH, Chang HS, Chung WY, Park CS. Robotic thyroid surgery using a gasless, transaxillary approach and the da Vinci S system: the operative outcomes of 338 consecutive patients. Surgery. 2009 Dec;146(6):1048-55
e87. Richmon JD, Holsinger FC, Kandil E, Moore MW, Armando Garcia J, Tufano RP. Transoral robotic-assisted thyroidectomy with central neck dissection: preclinical cadaver feasibility study and proposed surgical technique. J Robot Surg. 2011 Dec;5(4):279-82
e88. Kim SK, Woo JW, Park I, Lee JH, Choe JH, Kim JH, Kim JS. Propensity score-matched analysis of robotic versus endoscopic bilateral axillo-breast approach (BABA) thyroidectomy in papillary thyroid carcinoma. Langenbecks Arch Surg. 2017 Mar;402(2):243-250
e89. Radford PD, Ferguson MS, Magill JC, Karthikesalingham AP, Alusi G. Meta-analysis of minimally invasive video-assisted thyroidectomy. Laryngoscope. 2011 Aug;121(8):1675-81
e90. Landry CS, Grubbs EG, Morris GS, Turner NS, Holsinger FC, Lee JE, Perrier ND. Robot assisted transaxillary surgery (RATS) for the removal of thyroid and parathyroid glands. Surgery. 2011 Apr;149(4):549-55
e91. Eckhardt S, Maurer E, Fendrich V, Bartsch DK. Transaxilläre roboterassistierte Schilddrüsenresektion. Chirurg. 2015 Oct;86(10):976-82
e92. Maurer E, Wächter S, Albers M, Holzer K, Bartsch DK. Die transaxilläre roboterassistierte Thyreoidektomie – Ergebnisse und Akzeptanz einer neuen Operationsmethode. Zentralbl Chir 2018; 143(04): 353-360
e93. Karakas E, Klein G, Schopf S. Transoral thyroid surgery vestibular approach. Innov Surg Sci. 2022 Jun 17;7(3-4):107-113
e94. Bollerslev J, Rejnmark L, Marcocci C, M Shoback DM, Sitges-Serra A, van Biesen W, OM; European Society of Endocrinology. European Society of Endocrinology Clinical Guideline: Treatment of chronic hypoparathyroidism in adults. Eur J Endocrinol. 2015 Aug;173(2):G1-20
e95. Bilezikian JP, Brandi ML, Cusano NE, Mannstadt M, Rejnmark L, Rizzoli R, Rubin MR, Winer KK, Liberman UA, Potts Jr JT. Management of Hypoparathyroidism: Present and Future. J Clin Endocrinol Metab. 2016 Jun;101(6):2313-24
